# Expansion of Genes Encoding piRNA-Associated Argonaute Proteins in the Pea Aphid: Diversification of Expression Profiles in Different Plastic Morphs

**DOI:** 10.1371/journal.pone.0028051

**Published:** 2011-12-05

**Authors:** Hsiao-ling Lu, Sylvie Tanguy, Claude Rispe, Jean-Pierre Gauthier, Tom Walsh, Karl Gordon, Owain Edwards, Denis Tagu, Chun-che Chang, Stéphanie Jaubert-Possamai

**Affiliations:** 1 Department of Entomology/Institute of Biotechnology, College of Bioresources and Agriculture, National Taiwan University, Taipei, Taiwan; 2 UMR (Unité Mixte de Recherche) 1099 BiO3P (Biologie des Organismes et des Population appliquée à la Protection des Plantes) INRA (Institut National de la Rechercher Agronomique) – Agrocampus – Université Rennes1, Le Rheu, France; 3 CSIRO (Commonwealth Scientific and Industrial Research Organisation) Ecosystem Sciences, Canberra, Australia; 4 CSIRO Ecosystem Sciences, Wembley, Australia; 5 Research Centre for Developmental Biology and Regenerative Medicine, National Taiwan University, Taipei, Taiwan; 6 Genome and Systems Biology Degree Program, National Taiwan University, Taipei, Taiwan; Ghent University, Belgium

## Abstract

Piwi-interacting RNAs (piRNAs) are known to regulate transposon activity in germ cells of several animal models that propagate sexually. However, the role of piRNAs during asexual reproduction remains almost unknown. Aphids that can alternate sexual and asexual reproduction cycles in response to seasonal changes of photoperiod provide a unique opportunity to study piRNAs and the piRNA pathway in both reproductive modes. Taking advantage of the recently sequenced genome of the pea aphid *Acyrthosiphon pisum*, we found an unusually large lineage-specific expansion of genes encoding the Piwi sub-clade of Argonaute proteins. *In situ* hybridisation showed differential expressions between the duplicated *piwi* copies: while *Api-piwi2* and *Api-piwi6* are “specialised” in germ cells their most closely related copy, respectively *Api-piwi5* and *Api-piwi3*, are expressed in the somatic cells. The differential expression was also identified in duplicated *ago3*: *Api-ago3a* in germ cells and *Api-ago3b* in somatic cells. Moreover, analyses of expression profiles of the expanded *piwi* and *ago3* genes by semi-quantitative RT-PCR showed that expressions varied according to the reproductive types. These specific expression patterns suggest that expanded aphid *piwi* and *ago3* genes have distinct roles in asexual and sexual reproduction.

## Introduction

Small non-coding RNAs play key roles in the regulation of gene expression and control of transposon activity in eukaryotes. These RNAs include microRNAs (miRNAs) that regulate gene expression, endogenous small interfering RNAs (endo siRNAs) that regulate gene expression and transposition in somatic cells, and Piwi-interacting RNAs (piRNAs) that silence transposons in germ cells. They are key components of the various small RNA silencing pathways and act within protein complexes including the Argonaute proteins, which represent a large protein family with different small RNA specificities [1]. All members of the Argonaute family share three structural motifs: the Piwi-Argonaute-Zwille (PAZ) domain, the Middle (MID) domain, and the PIWI domain. The function of the PAZ domain is not known but it can bind to the characteristic 2-base 3′ overhangs of small RNAs (sRNAs) digested by RNase III. The MID and PIWI domains, on the other hand, are associated with the 5′ ends of the small RNAs. Among these three domains, PIWI contains the catalytic DDH triad composed of two Aspartic Acid residues (D) and one Histidine (H) residue and responsible for the RNAseH-like endonuclease activity. It can trigger the double-stranded RNA (dsRNA) guided hydrolysis of single-stranded RNA (ssRNA) and it possesses the endonuclease “slicer” activity [2], [3].

*Argonaute* genes are highly conserved but their numbers vary between organisms ranging from one copy in the fission yeast *Schizosaccharomyces pombe* [4], to five in the fruit fly *Drosophila melanogaster* [5], and 27 in the nematode *Caenorhabditis elegans* [6]. The Argonaute protein family is subdivided into two subgroups, the Ago subfamily and the Piwi subfamily, according to their distinct functions and small RNA specificities. The Ago subfamily binds to small interfering RNAs (siRNAs) as well as miRNAs, playing a central role in siRNA and miRNA silencing pathway; the Piwi subfamily is involved in the piRNA pathway, participating in germline development, meiosis and gametogenesis [7] but their role in somatic tissues remains unclear [7], [8]. In *Drosophila*, the Ago subfamily is composed of the miRNA-related Argonaute-1 (Ago1) and the siRNA-related Argonaute-2 (Ago2) whilst the Piwi subfamily, which includes Aubergine (Aub), Piwi and Argonaute-3 (Ago3), is involved in the piRNA pathways [9]. Usually expression of the Piwi subfamily is restricted to the germ cells while that of the Ago subfamily is ubiquitous. However, this scenario has been questioned since *Drosophila* Piwi has been recently identified in both the germline and the ovarian somatic support cells [10]–[12], in contrast to Ago3 and Aub that are restricted to the germline [13], [14].

To date, piRNAs in *Drosophila* can be categorized as two distinct types: the germline piRNAs and the somatic piRNAs. The germline piRNAs, which are associated with Aub, Piwi and Ago3, are processed by the “ping-pong mechanism”. The somatic piRNAs, which are usually associated only with Piwi, are generated via the “primary piRNA model” [7], [15]. It has been demonstrated that the germline piRNAs are involved in germline specification, maintenance of germ cells, meiosis and transposon movement [7]. In addition, roles for piRNAs in early embryogenesis have been described for the Piwi subfamily and piRNAs in *Drosophila* [16], [17] and zebrafish [18]–[21]. In *Drosophila*, maternally inherited piRNAs are present in early embryos where they provide resistance to transposition [16]. Moreover, Aub and Ago3, both of which are components of the piRNA pathway, are found to associate with Smaug and the CCR4 deadenylase complex to regulate the decay of the maternal *nanos* mRNA, a posterior determinant in *Drosophila* [17]. In zebrafish, both *ziwi* and *zili* encode Piwi proteins that can bind piRNAs of opposite polarity and suppress the transposon activity in germ cells [18], [19]. In addition to these conserved roles in transposon defense and germline development, recent reports show that *zili* is involve in axis patterning via transforming growth factor (TGF)-β and fibroblast growth factor (FGF) signaling pathways [20], [21].

Here, we provide information on the piRNA pathway for the pea aphid *Acyrthosiphon pisum*, an emerging genomic model organism with a recently sequenced and annotated genome [22]. This phloem-sucking insect has an unusual capacity for phenotypic plasticity, displaying an ability to adapt its phenotype according to environmental conditions [23]. Aphids switch their reproductive mode in response to seasonal changes. During spring and summer, aphids reproduce by clonal viviparous parthenogenesis: a single female can give birth to approximately 80 larvae in 10 days, all of which are genetically identical to each other and to their mother. This reproductive mode allows a rapid and effective colonisation of host plants during the growing seasons. In autumn, the decrease of day length induces parthenogenetic viviparous females (named sexuparae) that produce sexual males and sexual oviparous females (named oviparae). Embryos in both virginoparae and sexuparae viviparous females complete development within the mother, and prior to larviposition germ cells within the embryos are specified and migrate to coalesce with the somatic gonads [24] – resulting in a phenomenon known as “telescoping of generations”. The sexual males and oviparous sexual females then mate and produce overwintering eggs [25]. The pea aphid thus offers a great opportunity to analyse the molecular mechanisms of sexual and asexual reproduction, including the roles of small non-coding RNAs in the alternation of reproductive modes and in the development of the different sexual and asexual morphs. We describe here an expansion of the piRNA associated Argonaute family in the pea aphid. This expansion correlates to a diversification of developmental and spatial expression profiles in different reproductive morphs. Diversified expressions of these expanded copies suggest an adaptation of the piRNA pathways to contribute to the regulation of reproductive plasticity in aphids.

## Results

### Piwi-type Argonautes in the pea aphid genome

Aphid homologues of genes from the Piwi subgroup of the Argonaute family (Piwi and Ago3) were identified in the *A. pisum* genome. All of the eight Api-Piwi and the two Api-Ago3 deduced proteins possess the functional PAZ, MID and PIWI domains (Data S1) characteristic of the Argonaute family. The main residues involved in the binding of the 5′ end of small RNAs and the DDH catalytic triad are conserved in most of the *A. pisum* proteins, suggesting that these proteins are functional Argonautes. However, the mutation of the DDH triad into DDR in Api-Piwi8 indicates that this protein may have lost slicer activity (Data S1).

**Figure 1:**
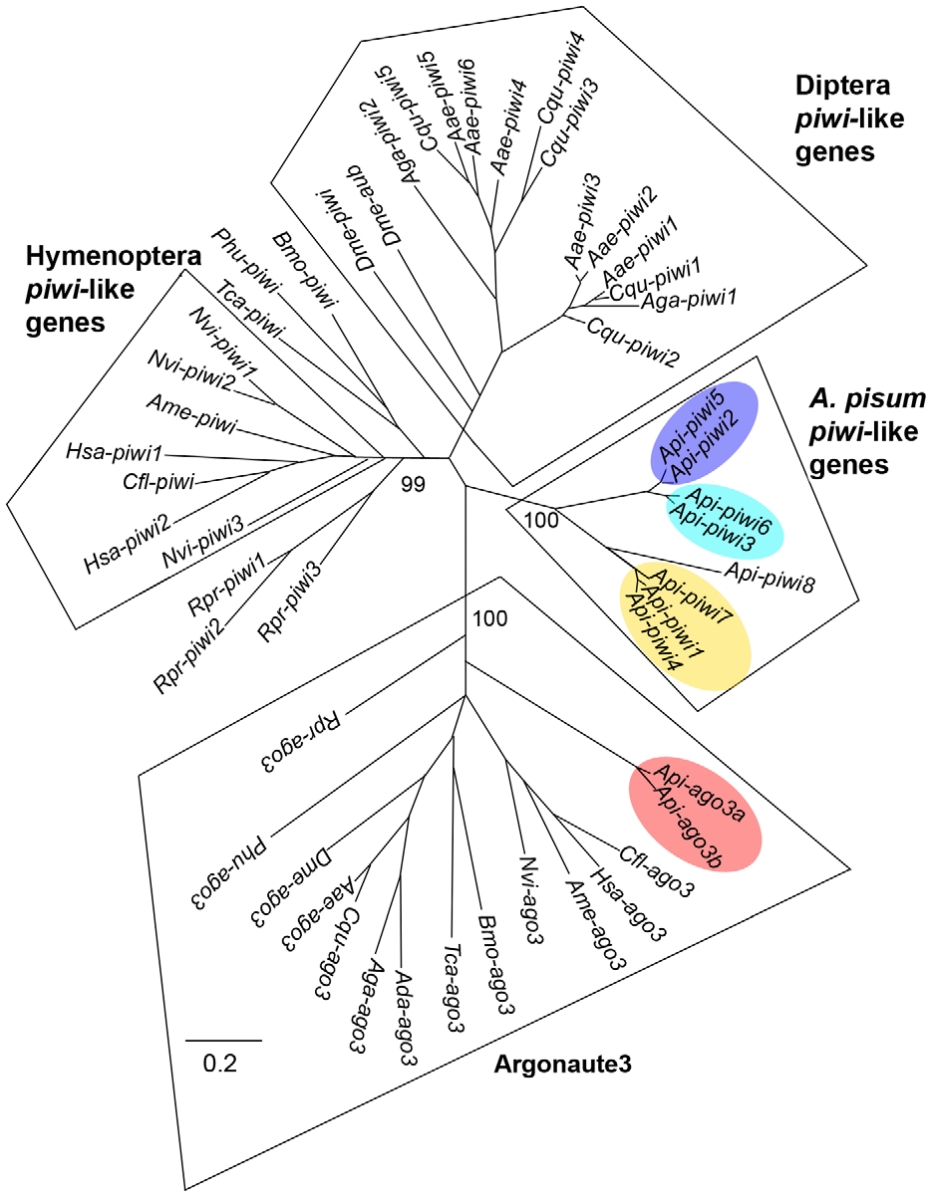
ML phylogenetic tree including protein Piwi-like and Ago3-like sequences from insects. Piwi-like and Ago3-like sequences were retrieved from insect species with a complete genome sequence. Prefixes correspond to abbreviated species names: *Aae*, *Aedes aegypti*; *Ada*, *Anopheles darlingii*; *Aga*, *Anopheles gambiae*; *Ame*, *Apis mellifera*; *Api*, *Acyrthosiphon pisum*; *Bmo*, *Bombyx mori*; *Cfl*, *Camponotus floridanus*; *Cqu*, *Culex quinquefasciatus*; *Dme*, *Drosophila melanogaster*; *Hsa*, *Harpegnathos saltator*; *Nvi*, *Nasonia vitripennis*; *Phu*, *Pediculus humanus*; *Rpr*, *Rhodnius prolixus*; *Tca*, *Tribolium castaneum*. Gene accession numbers are provided in Table S2. Bootstrap values are shown only for the most relevant groups discussed in the Materials and Methods section.

In addition, we identified *piwi* homologues in the genome of another hemipteran, the blood-sucking bug *Rhodnius prolixus*. The genome of *R. prolixus* has been recently sequenced (http://genome.wustl.edu/genomes/view/rhodnius_prolixus/) and it is to date the only sequenced genome in the same order (Hemiptera) as the pea aphid. Different phylogenetic methods were tested (neighbor-joining (NJ) and maximum likelihood (ML) with different substitution models) and gave the same results (Figure 1 represents the ML tree with the BLOSUM62 model). First, a group of Ago3-like sequences was clearly identified, as it consists of a robust group with 100% bootstrap support. Two complete copies (*Api-ago3a* and *Api-ago3b*) were identified in the pea aphid genome. Interestingly, all other insect genomes surveyed contain a single *ago3* gene. In fact, the pea aphid was even found to contain a third copy *Api-ago3c* very close to *Api-ago3b*, but this third copy appears to be incomplete and contains in-frame stop codons, suggesting that it is a pseudogene (this sequence was thus discarded and not included in the analysis). When compared to *Api-ago3a*, *Api-ago3b* appears to evolve more rapidly as shown by its longer branch. For *piwi*-like genes, the *A. pisum* genome was found to contain eight complete copies (*Api-piwi1* to *8*) -three further *piwi*-like genes were detected; however, like *Api-ago3c* these were incomplete, having in-frame stop codons and frameshifts, and were thus considered as likely pseudogenes. *Piwi*-like genes from *A. pisum* made a robust phylogenetic group, showing that the different copies arose though lineage-specific duplication. This gene family is divided in two subgroups (*Api-piwi2*, *3*, *5*, *6* versus *Api-piwi1*, *4*, *7*, *8*), which appear to correspond to a relatively ancient duplication event (Figure 1). More recent duplications would have followed, defining more recent groups of paralogues (*Api-piwi2 and 5, Api-piwi3 and 6, and Api-piwi 1,4,7)*. These closely related paralogues are particularly interesting to compare in terms of their expression profile, to determine the pace of specialisation or sub-functionalisation processes.

Gene amplifications can also be seen in representatives of the insect orders Hymenoptera (for *Nasonia vitripennis* and *Harpegnathos saltator*) and Diptera (for *D. melanogaster*, *Anopheles gambiae*, *Culex quinquefasciatus*, and *Aedes aegypti*); nevertheless, *A. pisum* shows the largest *piwi* expansion for any insect investigated to date. Within the Culicidae (mosquitoes), the copies can be divided in two subgroups that probably correspond to a relatively ancient duplication preceding speciation. In *R. prolixus*, the other hemipteran insect included in this analysis, genomic amplification was also detected, resulting in 3 copies (*Rpr-piwi1* to *3*). However this amplification was found to be independent of the *A. pisum* duplications. Interestingly, *Rpr-piwi1* and *Rpr-piwi2* are intronless and might have arisen through one retroposition event (followed by duplication), and the longer branches (in particular for *Rpr-piwi2*) suggest accelerated evolutionary rates for these two copies. By contrast, none of the *A. pisum* copies are intronless.

### Expression profiles in the reproductive morphs

**Figure 2:**
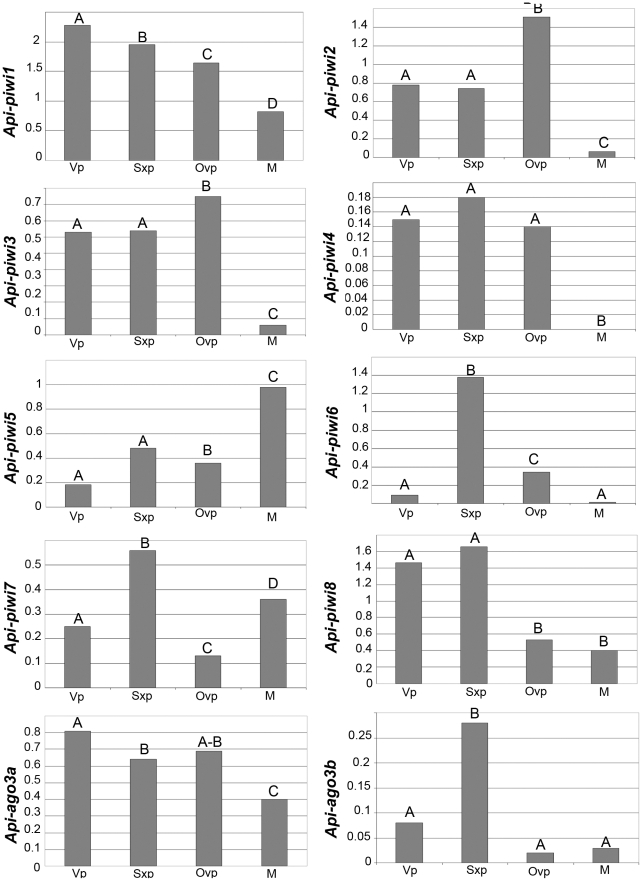
Expression profiles of *Api-piwi* and *Api-ago3* genes of *A. pisum* in the four reproductive morphs. Expression levels of the eight *Api-piwi* (1–8) and the two *Api-ago3* (a and b) transcripts were investigated by semi-quantitative RT-PCR on total RNA of adults collected 48 h after adult moult of the four reproductive morphs: parthenogenetic virginoparae females (Vp), parthenogenetic sexuparae females (Sxp), sexual oviparae females (Ovp) and sexual males (M) (See Figure S1 for gel photography). Expression of *Api-piwi* and *Api-ago3* genes in the different morphs was normalised against the gene encoding ribosomal protein 7 (*Api-rpl7*) as a reference gene [45]. The ratio between the expression of the amplified gene and of the *Api-rpl7* reference gene was calculated to normalise for variations in experimental conditions. For each morph, gene expression was measured from three batches of 10 adult aphids resulting from three independent biological experiments. For each gene, an average normalised expression level was calculated for each reproductive morph. In order to test the significance of variation of expression level, the data were subjected to arc-sine transformation to analyse variance. Letters indicate statistical significant similar or different expression levels in the samples.

Aphids display the amazing ability to switch from asexual parthenogenesis to sexual reproduction in response to seasonal modification of photoperiods. Expression level of the eight *Api-piwi* and two *Api-ago3* genes was analysed by semi-quantitative RT-PCR in the different reproductive morphs of the pea aphid (Figure S1): adult parthenogenetic virginoparae females (that produce parthenogenetic embryos), adult parthenogenetic sexuparae females (that produce sexual embryos), sexual oviparae females, and males. For each morph, RT-PCR was performed on RNA extracted from three independent batches of 10 adult whole bodies collected 48 h after adult moult. The ratio between the expression of the amplified gene and that of the *Api-rpl7* reference gene was calculated to normalise the variation in experimental conditions. An average expression level was calculated for each reproductive morph based on the expression level obtained from the three independent batches (Figure 2). ANOVA tests were performed on the normalised values for each reproductive morph.

All the eight *Api-piwi* and the two *Api-ago3* genes were expressed in females but showed distinct expression profiles that varied between the morphs (Figure 2). Most *Api-piwi* genes and *Api-ago3a* genes were expressed at a lower level in males than in the three female morphs. For example, *Api-piwi2*, *3*, *4* and *6* were almost undetectable in males. However, *Api-piwi5* was strongly expressed in males, and *Api-piwi7* expression level is stronger in males than in virginoparae and oviparae females. As for genes that were preferentially expressed in some morphs, the following specific expression patterns were noteworthy: (1) The expression profiles of *Api-piwi2* and *Api-piwi8* discriminated between parthenogenetic females (virginoparae and sexuparae) and sexual females (oviparae), with *Api-piwi2* clearly overexpressed in sexual females and *Api-piwi8* in asexuals; (2) *Api-piwi5* was preferentially expressed in males while it was only weakly expressed in the female morphs; (3) *Api-piwi*6 and *Api-ago3b* were preferentially overexpressed in sexuparae females. Taken together, these results indicate that the expansion of the piRNA-related *piwi* and *ago3* genes in the pea aphid appears to be linked to its reproductive plasticity.

### Developmental expression of *Api-piwi* and *Api-ago3* genes

The temporal and spatial distribution of the eight *Api-piwi* and two *Api-ago3* transcripts were analysed by *in situ* hybridisation on dissected ovarioles containing adult germaria, oocytes, and developing embryos. For each gene, specific antisense riboprobe was designed to detect the distribution of the corresponding transcripts; sense riboprobes were applied in negative controls. Because of the high sequence identity between *Api-piwi1*, *4* and *7* (>92%), we were not able to synthesise specific probes that could discriminate individual expressions of these three genes. Nevertheless, *in situ* signals detected by antisense riboprobes synthesised from different template regions of *Api-piwi1*, *4*, and *7* reflected consensus expression patterns.

**Figure 3:**
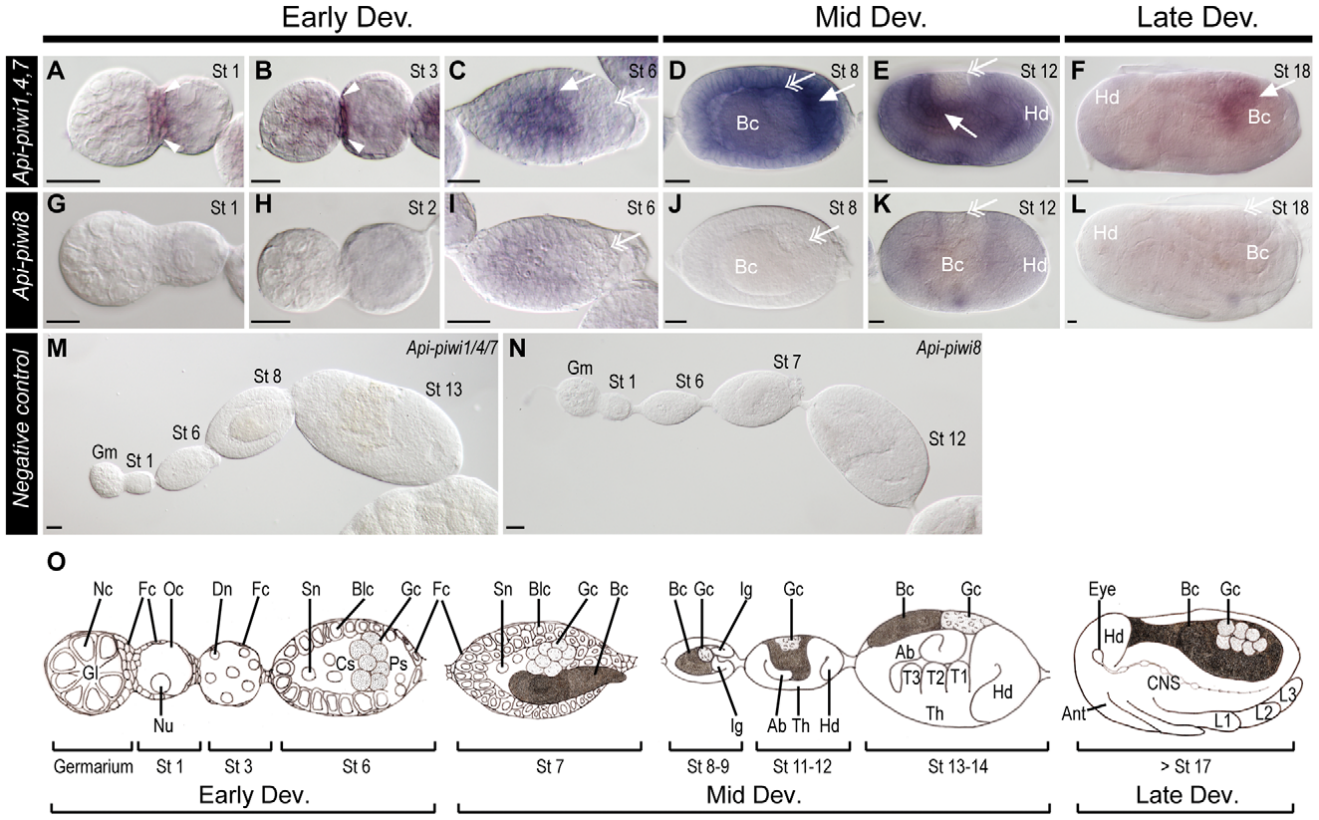
Comparison of developmental expressions of *Api-piwi1,4,7* and *Api-piwi8* in virginoparous embryos. Samples were hybridised with *Api-piwi1,4,7* (A–F) and *Api-piwi8* (G–L) antisense riboprobes. Anterior is to the left; dorsal is upper. All views are lateral. Unless addressed specifically, locations of germ cells are labelled with double arrowheads. (**A, G**) Germaria and segregated oocytes (stage 1). Preferential expression was identified in the follicle cells between the germarium and the oocyte (arrowheads). *Api-piwi8* transcripts were not detected. (**B, H**) Embryos undergoing nuclear divisions (stage 2, 3). *Api-piwi1,4,7* expression remained restricted to the follicle cells (arrowheads). *Api-piwi8* stayed undetected. (**C, I**) Embryos with newly-segregated germ cells (stage 6). Most *Api-piwi1,4,7* transcripts were restricted to the central syncytium (arrow), but *Api-piwi1,4,7* expression was not detected in germ cells. *Api-piwi8* was weakly expressed in anterior two thirds of the egg. (**D, J**) Embryos undergoing gastrulation (stage 8). *Api-piwi1,4,7* expression was preferentially identified in the invaginating germband (arrow). *Api-piwi8* expression was almost undetected. (**E, K**) Extension of the germband (stage 12). Strong expression of *Api-piwi1,4,7* was identified in the abdomen (arrow). Faint expression of *Api-piwi8* was visible. (**F, L**) Completion of germband retraction (stage 18). *Api-piwi1,4,7* transcripts were restricted to the gonadal germ cells (arrow); *Api-piwi8* expression was almost undetected. (**M, N**) Negative control, ovarioles hybridised with sense riboprobes. No signals were detected. (**O**) Illustration displaying presented developmental stages. Abbreviations: Ab, abdomen; Ant, antennae; Bc, endosymbiotic bacteria (invading embryos during stage 7); Blc, blastodermal cells; CNS; central nerve system; Cs, central syncytium (central blastoderm containing multiplying nuclei); Dev, development; Dn, dividing nuclei; Fc, follicle cells; Gc, germ cells; Gl, germarial lumen (central cavity of the germarium, where germline stem cells are derived); Gm, germarium; Hd, head; Ig, invaginating germband; L1-3, limbs 1–3; Nc, nurse cells; Nu, nuclei; Oc, oocyte; Ps, posterior syncytium; Sn, syncytial nuclei. St, stage; Th, thorax; T1-3, thoracic segments 1–3. Scale bar, 20 µm.

In virginoparae, combinational expression of *Api-piwi1*, *4* and *7* was detected in germaria, oocytes, and early embryos before gastrulation (Figure 3 A–C). Preferential expression was identified in the follicle cells located between germaria and oocytes (Figure 3A, B) but newly-segregated germ cells were almost devoid of staining (Figure 3C). During mid-embryogenesis, expression patterns appeared dynamic (Figure 3D, E), but *in situ* signals were preferentially detected in germ cells residing in the gonads of late embryos (Figure 3F). Expression of *Api-piwi8*, a gene within the same phylogenetic subgroup of *Api-piwi1*, *4*, and *7*, was almost undetected during oogenesis and embryogenesis (Figure 3 G–I) except in early embryos before gastrulation (Figure 3I). During mid and late embryogenesis, *Api-piwi8* was weakly expressed (Figure 3 J–L). Gene expression was not detected in ovarioles hybridised with sense riboprobes (Figure 3M, N), indicating that the *in situ* signals identified with antisense probes accurately represented the distributions of *Api-piwi1*, *4* and/or *7* and *Api-piwi8*. The developmental characteristics of embryos hybridised with probes of *Api-piwi1*, *4*, *7*, 8 and those stained with other gene probes are highlighted in the cartoon illustration shown in Figure 3O.

**Figure 4 pone-0028051-g004:**
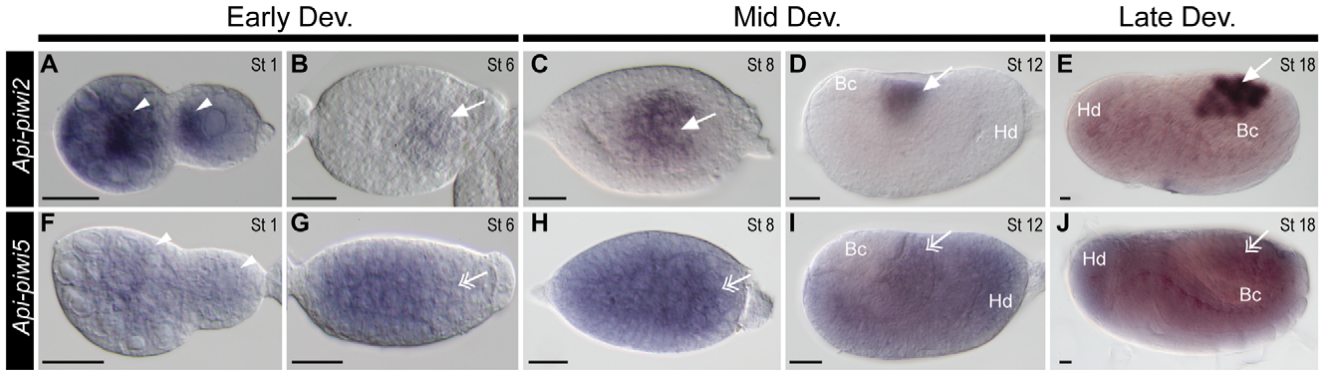
Comparison of expressions of *Api-piwi2* and *Api-piwi5* in the virginoparous embryos. Dissected ovarioles were hybridised with DIG-labelled antisense riboprobes of *Api-piwi2* (A–E) and *Api-piwi5* (F–J), respectively. Orientations and morphological characteristics of embryos refer to Figure 3O. Early development: A, B, F, G; Mid development: C, D, H, I; Late development: E, J. Germ cells stained with antisense *Api-piwi2* and *Api-piwi5* riboprobes are highlighted with arrows (with preferential expression) and double arrows (without preferential expression). (**A, F**) Germaria and segregated oocytes (stage 1). Expression of *Api-piwi2* was preferentially detected in the germarial lumen and the oocyte anterior (arrow heads). *Api-piwi5* mRNA was expressed comparatively weaker. (**B, G**) Embryos with newly-segregated germ cells (stage 6). Weak expression of *Api-piwi2* was specifically detected in the germ cells; expression of *Api-piwi5* was evenly distributed in anterior two thirds of the embryo and it was weakly detected in the germ cells. (**C, H**) Gastrulating embryos (stage 8). Like stage 6, expression of *Api-piwi2* was specifically detected in the germ cells but the intensity of *in situ* signals increased. Expression of *Api-piwi5* covered most region of the embryo including germ cells. Embryo in (H) is slightly younger than that in (C) so that germ cells have not been pulled into the middle region of the embryo. Bacteria are not clearly visible in the focal plane shown. (**D, I**) Extension of the germband (stage 12). Transcripts of *Api-piwi2* were specifically identified in the germ cells whilst transcripts of *Api-piwi5* were evenly distributed except in bacteria. (**E, J**) Completion of germband retraction (stage 18). Specific expression of *Api-piwi2* was identified in germ cells located in the dorsal region of the embryo, but universal expression of *Api-piwi5* remained as it was detected in the stage-12 embryos. Abbreviations: Bc, endosymbiotic bacteria; Dev, development; Hd, head; St, stage. Scale bar, 20 µm.

**Figure 5 pone-0028051-g005:**
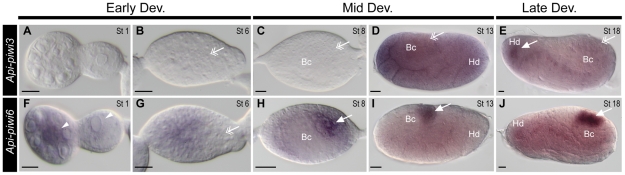
Comparison of expressions of *Api-piwi3* and *Api-piwi6* in virginoparous embryos. Dissected ovarioles were hybridised with DIG-labelled antisense riboprobes of *Api-piwi3* (A–E) and *Api-piwi6* (F–J). For orientations and morphological characteristics of embryos refer to Figure 3O. Early development: A, B, F, G; Mid development: C, D, H, I; Late development: E, J. Germ cells stained with antisense *Api-piwi3* and *Api-piwi6* riboprobes are indicated by arrows (with preferential expression) and double arrows (without preferential expression). (**A, F**) Germaria and segregated oocytes (stage 1). Expression of *Api-piwi3* was not detected but preferential expression of *Api-piwi6* could be identified in the germarial lumen (arrowheads). Weak expression of *Api-piwi6* was visible in the cytoplasm of the oocyte. (**B, G**) Embryos with newly-segregated germ cells (stage 6). Expression of *Api-piwi3* was not detected in germ cells. Transcripts of *Api-piwi6* were evenly distributed in the anterior two thirds of the egg chamber but germ cells were devoid of staining. (**C, H**) Gastrulating embryos (stage 8). Expression of *Api-piwi3* was not detected in germ cells and other places in the egg chambers. Specific expression of *Api-piwi6* could be identified in the germ cells. (**D, I**) Limb bud formation (stage 13). Expression of *Api-piwi3* was evenly distributed in the embryo. Preferential expression of *Api-piwi6* was restricted to germ cells in the dorsal region and weak expression was identified in other regions of the embryo. (**E, J**) Completion of germband retraction (stage 18). Expression of *Api-piwi3* was evenly distributed in the embryo but preferential expression was indentified in the region of the head (arrow). Expression of *Api-piwi6* remained detected specifically in germ cells. Abbreviations: Bc, endosymbiotic bacteria; Dev, development; Hd, head; St, stage. Scale bar, 20 µm.

Coding regions with more than 25% sequence difference were used as templates to synthesise probes to distinguish expression of the closely related *Api-piwi2/Api-piwi5* and *Api-piwi3/Api-piwi6* genes in virginoparae embryos. During early development, transcripts of *Api-piwi2* were restricted to the germarial lumen and the anterior region of the oocytes (Figure 4A). Weak expression of *Api-piwi2* was visible in the newly-segregated germ cells (Figure 4B), but strong intensity of the germline-specific signals of *Api-piwi2* were identified from gastrulation onward (Figure 4 C–E). In comparison with *Api-piwi2*, a lower level of *Api-piwi5* transcript abundance was identified in germaria and oocytes (Figure 4F). Expression of *Api-piwi5* was evenly distributed in embryos throughout development and no preferential expression was identified in germ cells (Figure 4 G–J). Expression of *Api-piwi3* was undetected in germaria and gastrulating embryos (Figure 5 A–C), and transcripts of *Api-piwi3* appeared not specifically restricted to germ cells (Figure 5D, E). In contrast, the transcripts of its closely related copy *Api-piwi6* were identified specifically in germ cells from mid embryogenesis onward (Figure 5 H–J) as was also observed for *Api-piwi2* (Figure 4 C–E), another germline-specific *piwi* gene in the pea aphid. However, preferential expression of *Api-piwi6* was detected neither in the oocyte anterior (Figure 5F) nor in the newly-segregated germ cells (Figure 5G), which distinguished its expression pattern from that of *Api-piwi2* (Figure 4A, B) during early embryogenesis.

**Figure 6:**
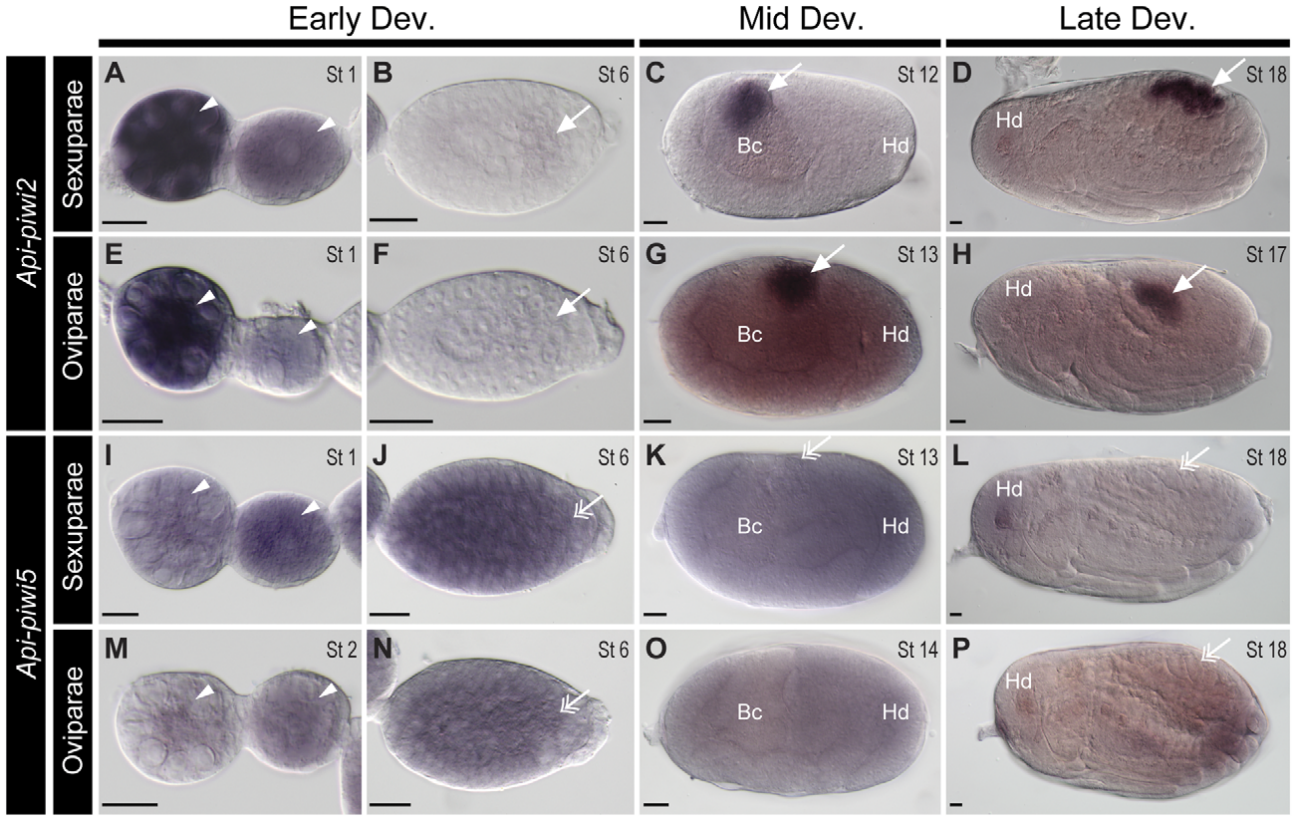
Comparison of expressions of *Api-piwi2* and *Api-piwi5* in sexuparous and oviparous embryos. Ovarioles were dissected from adult virginoparae raised under short day conditions that induce the production of sexuparae, and from adult sexuparae that produce sexual oviparae females and males. Ovarioles were hybridised with DIG-labelled antisense riboprobes of *Api-piwi2* (A–H) and *Api-piwi5* (I–P), respectively. For orientations and morphological characteristics of embryos refer to Figure 3O. Early development: A, B, E, F, I, J, M, N; Mid development: C, G, K, O; Late development: D, H, L, P. Germ cells stained with antisense *Api-piwi2* and *Api-piwi5* riboprobes are highlighted with arrows (with preferential expression) and double arrows (without preferential expression). (**A, E, I, M**) Germaria and segregated oocytes (stage 1). Transcripts of *Api-piwi2* were detected in the germaria and the oocyte (arrowheads). Expression of *Api-piwi5* remained the same pattern as that of *Api-piwi2*, but signal intensity in germaria was reduced. (**B, F, J, N**) Embryos with newly-segregated germ cells (stage 6). Weak expression of *Api-piwi2* was specifically detected in the germ cells; expression of *Api-piwi5* was evenly distributed in the embryo including the germ cells. (**C, G, K, O**) Extension of the germband and limb bud formation (stage 12-14). Transcripts of *Api-piwi2* were preferentially identified in the germ cells of sexuparae and oviparae embryos but expression of *Api-piwi5* was evenly distributed. In panel (O), germ cells are not presented in the shown focal plane. (**D, H, L, P**) Germband retraction (stage 17) and completion of germband retraction (stage 18). Specific expression of *Api-piwi2* was identified in germ cells located in the dorsal region of the embryo, but germline-specific expression of *Api-piwi5* was not detected. Abbreviations: Bc, endosymbiotic bacteria; Dev, development; Hd, head; St, stage. Scale bar, 20 µm.

Localisation of these transcripts was also investigated in ovarioles containing the embryonic stages of parthenogenetic sexuparae and sexual oviparae. The expression patterns of *Api-piwi* genes in sexuparae and oviparae were similar to those obtained from virginoparae: *Api-piwi2* and *Api-piwi6* were germline-specific while their closely related copies *Api-piwi5* and *Api-piwi3* respectively were not (Figure 6 and Figure S2). However, *Api-piwi6* showed a distinct localisation of expression between asexual and sexual embryos: it was preferentially restricted to germ cells in the virginoparae (Figure 5 H–J) and sexuparae (Figure S2K, L), but it was evenly expressed in the oviparae (Figure S2O, P).

**Figure 7:**
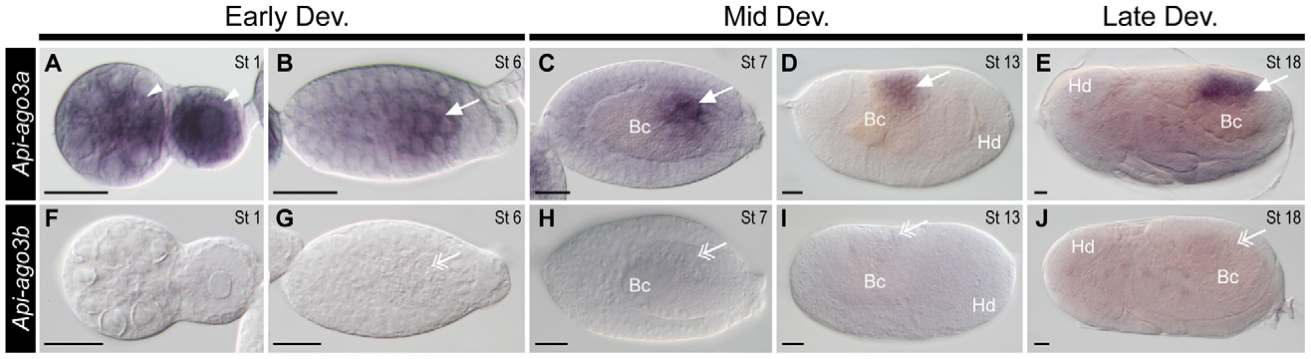
Comparison of expressions of *Api-ago3a* and *Api-ago3b* in virginoparous embryos. Dissected ovarioles were hybridised with DIG-labelled antisense riboprobes of *Api-ago3a* (A–E) and *Api-ago3b* (F–J). For orientation and morphological characteristics of embryos refer to Figure 3O. Early development: A, B, F, G; Mid development: C, D, H, I; Late development: E, J. Germ cells stained with antisense *Api-ago3a* and *Api-ago3b* riboprobes are highlighted with arrows (with preferential expression) and double arrows (without preferential expression). (**A**) Expression of *Api-ago3a* was identified in the germarial lumen and the cytoplasm of the segregated oocytes (arrowheads). (**B–E**) From stage 6 onward, specific expression of *Api-ago3a* was detected in germ cells. (**F–J**) Expression of *Api-ago3b* was not detected in germaria (F), segregated oocytes (F) and embryos (G–J). Background staining in late embryos (J) was also seen in embryos hybridised with sense riboprobe of *Api-ago3b* (data not shown). Abbreviations: Bc, endosymbiotic bacteria; Dev, development; Hd, head; St, stage. Scale bar, 20 µm.

In addition to the *piwi* genes, we also analysed the expression of *ago3* genes (*Api-ago3a* and *Api-ago3b*) in the pea aphid. In virginoparae, *Api-ago3a* was expressed in the germarial lumen as well as in the cytoplasm of oocytes (Figure 7A). During embryogenesis, the transcripts of *Api-ago3a* were preferentially restricted to the primordial germ cells (Figure 7B), migrating germ cells (Figure 7C, D), and germ cells in the gonads (Figure 7E). Expression of *Api-ago3b* was not detected in the germ cells of developing virginoparae, nor was it detected in any other cells throughout development (Figure 7 F–J). In strong contrast to the virginoparae, expression of *Api-ago3a* in sexuparae and oviparae was not restricted to the germ cells (Figure S3 A–H), but expression of *Api-ago3b* remained almost undetected (Figure S3 I–P).

In order to verify the expression of *Api-piwi2*, *Api-piwi6* and *Api-ago3a* are located in the embryonic germ cells, we performed double *in situ* hybridisation of these three genes with the germline marker gene *vasa* [24]. Experimental results show that transcripts of *Api-piwi2*, *Api-piwi6* and *Api-ago3a* are colocalised with *vasa* to the germ cells (Figures S4, S5, S6), confirming that their preferential expressions are restricted to the germline.

## Discussion

### Evolutionary diversification of *Api-piwi* genes

We describe here an expansion of the genes encoding Piwi sub-clade of Argonaute proteins in the pea aphid *A. pisum*. Annotation of the *A. pisum* genome identified eight copies of the *piwi* genes and two copies of the *Api-ago3*. Conservation of the key residues of PAZ, MID and PIWI domains suggest that Api-Piwi1-7 proteins are functional Argonautes while Api-Piwi8 presented mutation within the DDH catalytic triad and may have lost slicer activity. Since *Api-piwi8* results from an ancient duplication, this mutation appears to be evolutionary conserved. From the comparison of complete genomes in different insect orders, the duplication of *ago3* in aphids appears to be unique (Figure 1). By contrast, duplications of *piwi* have been reported in several organisms: at least six *piwi* genes have been identified in the mosquitoes *Culex pipiens* and *A. aegypti* respectively [26]; six *piwi* genes have been annotated in the genome of the crustacean *Daphnia pulex* [27]; 12 and 15 *piwi* genes have been characterized in the ciliate protozoa *Tetrahymena thermophila* and *Paramecium tetraurelia* respectively [28]–[30]. The biological significance of the expansions in mosquitoes and *Daphnia* has not yet been examined in detail. However, in the protozoan *T. thermophila* and *P. tetraurelia*, the expansion of *piwi* genes has been associated with neo- or sub-functionalisation of these proteins [28]–[30], as has been observed previously for Argonaute proteins in plants [31] and in the nematode *C. elegans* [6]. In most cases, including the pea aphid, *piwi* expansions appear to be independent events and lineage-specific, as shown by the monophyletic grouping of copies from each species. Indeed, the pea aphid expansion is not only independent of the expansion in mosquitoes, but also of the expansion we identified in its closer relative *R. prolixus*. Thus the expansions of the Piwi family in different animal groups appear to represent the repeated unfolding of a similar scenario.

### Diversification of developmental expression profiles in germline and in soma

**Figure 8:**
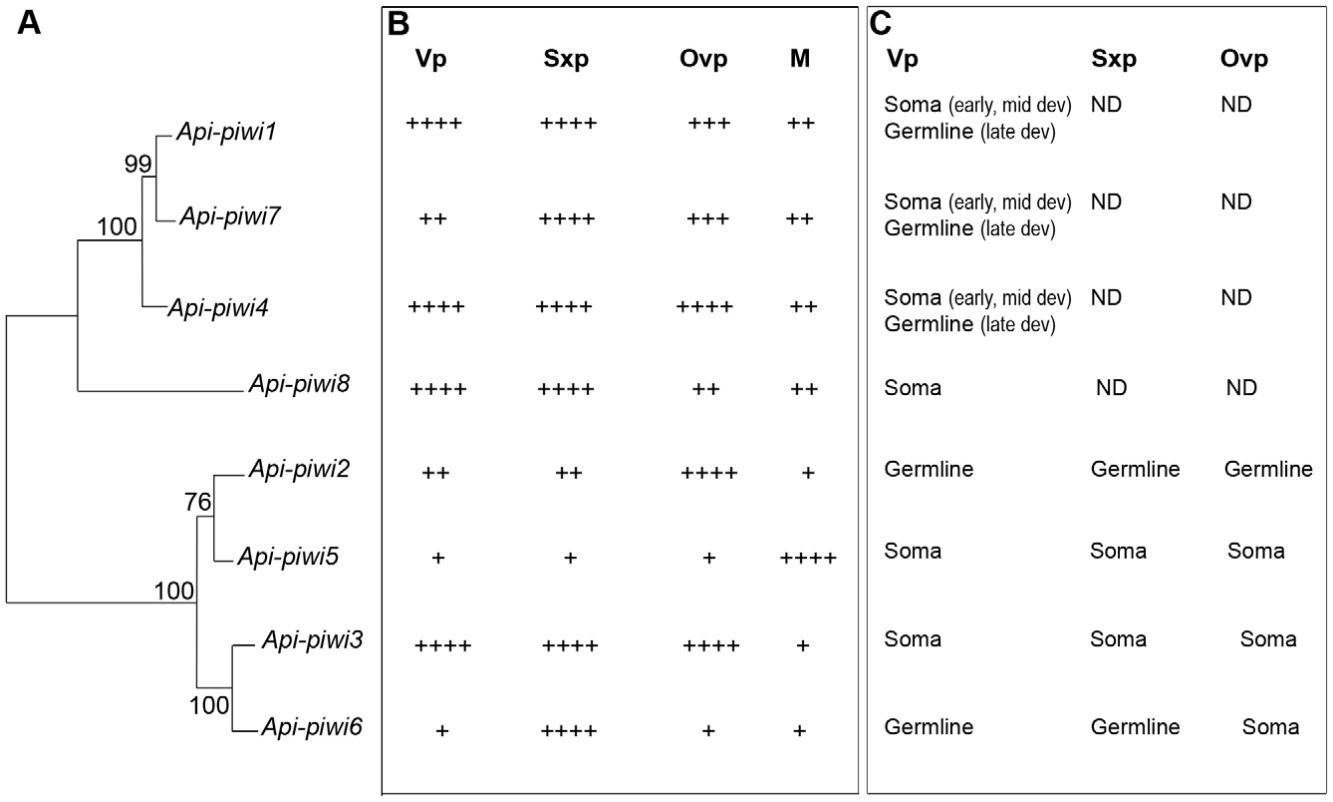
Summary of *piwi* expressions in the pea aphid. Integration of phylogenetic, semi-quantitative RT-PCR and *in situ* hybridisation analyses on the expanded *piwi* genes of the pea aphid. **A**: NJ tree of *Api-piwi* genes; boostrap values are indicated. **B**: Expression level of *Api-piwi* genes measured by semi-quantitative RT-PCR on the four reproductive morphs: female virginoparae (Vp), female sexuparae (Sxp), female oviparae (Ovp) and males (M) (see Figure 2 and Figure S1). Expression level was represented by “+”.**C**: Germline/soma expressions of *Api-piwi* genes during embryogenesis (see Figures 3–6 and Figure S2). Expressions of *Api-piwi1,4,7* and *Api-piwi8* were not determined (ND) in sexuparous and oviparous embryos. Localisation of transcripts was not determined in males since *in situ* techniques have not been applicable to this morph yet. Abbreviation: Dev., development.

*In situ* hybridisation on whole mounted ovarioles showed differential expression among some pea aphid *Api-piwi* and *Api-ago3* genes (*piwi* expression profiles are summarised in Figure 8). In animals, expression of Piwi and Ago3 is mainly restricted to the germline [19], [32], [33] and to the gonadal somatic cells in *Drosophila* [13]. In pea aphid virginoparae, we observed differential expression of the closely-related *piwi* genes *Api-piwi2/Api-piwi5* (Figure 4) and *Api-piwi3/Api-piwi6* (Figure 5), suggesting that duplication of *piwi* genes in this instance has led to the differentiation of *piwi* function between germline (*Api-piwi2*, *Api-piwi6*) and soma (*Api-piwi3*, *Api-piwi5*). Similar diversification of expression patterns of *piwi* paralogs has been reported in other organisms such as the ciliate protozoan *T. thermophila* or the crustacean water flea *D. pulex* [27], [29].

In *Drosophila*, the *piwi* gene family is represented by two copies, named respectively *piwi* and *aub*, also resulting from a duplication event. Like expanded *piwi* genes in aphids, *piwi* and *aub* show somatic/germline diversification of expression profiles in *Drosophila*: Piwi is expressed in somatic follicle cells as well as in germline while Aub and Ago3 are restricted to the germline [10], [13], [14]. These two spatial expression patterns are associated with two distinct functions. In ovarian somatic cells Piwi alone is involved in primary linear piRNA biogenesis, but in germline Piwi, Aub and Ago3 are involved in a more complex piRNA biogenesis pathway - the “ping-pong mechanism” that generates repeat-associated piRNAs in germ cells [7], [13], [15]. Taken together, it suggests that a common scenario occurs repetitively following duplication of *piwi* genes in distantly related organisms including the aphids: some copies are “specialised” in germ cells whereas others are expressed in the somatic cells.

Somatic expression was reported for *Api-piwi3* and *Api-piwi5* in the embryonic stages of the three female morphs, *Api-piwi6* in embryonic oviparae, and *Api-ago3a* in embryonic oviparae and sexuparae. Ubiquitous expression of *piwi*, *aub* and *ago3* has also been observed during *Drosophila* embryogenesis [5]. However this somatic expression in *Drosophila* was restricted to early embryos, in which a significant proportion of mRNA is maternally inherited [10]. In aphid embryos, somatic signals were observed for *Api-piwi5*, *Api-ago3a* and *Api-piwi6* in early and late embryo stages and specifically in late embryo stages for *Api-piwi6*. While signal observed until embryo blastula stage 6 can be imputed to maternal mRNAs [34], clear signal observed for these genes in late somatic stage (stage 12–18) reflects *de novo* somatic transcription (Table S1). In adult *Drosophila*, until recently, somatic expression of Piwi and piRNAs has only been reported in a specific type of somatic tissues: ovarian follicle cells, where they are responsible for the maintenance of germline stem cells (GSCs) in ovaries [15], [35]. Restriction of the *Api-piwi1,4,7* transcripts to the follicle cells in the posterior margin of the germarium (Figure 3A, B), from which the GSCs are derived [36], hence appears to suggest a potential role in the development of GSCs, like that in *Drosophila*. Apart from the ovarian follicle cells, recent findings show that piRNA-like molecules have now been identified in head and imaginal discs of *Drosophila* [8], but the biogenesis of the “non-ovarian follicle” somatic piRNAs still needs to be understood. Similarly, it remains to be solved whether the somatic ubiquitous zygotic expression of *Api-piwi3*, *Api-piwi5*, *Api-piwi6* in oviparae and *Api-ago3a* in sexuparae and oviparae reflects somatic expression in adults, as has been reported for several *piwi* genes in *D. pulex* [27]. Semi-quantitative RT-PCR realised on adult virginoparae heads did not evidence clear somatic expression of *Api-piwi3* and *Api-piwi5* (S. Jaubert-Possamai unpublished results). The role of such somatic *piwi* genes therefore requires further investigation.

### Diversification of expression profiles is correlated with the reproductive mode

In response to seasonal modifications of photoperiod, aphids can switch from asexual parthenogenesis to sexual reproduction. Analyses of expression profiles of the expanded *Api-piwi* and *Api-ago3* genes by semi-quantitative RT-PCR showed that these genes are differentially expressed in the various adult reproductive morphs of the pea aphid (*piwi* expression profiles are summarised in Figure 8). Every *Api-piwi* and *Api-ago3* gene exhibits a distinct expression profile, including the closely-related genes *Api-piwi2/Api-piwi5*, *Api-piwi3/Api-piwi6*, and *Api-ago3a/Api-ago3b*. These specific expression patterns of mRNAs suggest that expanded aphid Piwi proteins may have distinct roles in asexual or sexual reproductive phases.

*In situ* hybridisation also provided evidence for differentiation among reproductive morphs in the spatial expression of duplicated *Api-piwi* and *Api-ago3* genes. Interestingly, among all studied *argonaute* genes in the pea aphid, *Api-piwi2* is the only one that remains germline-specific in three different reproduction morphs (Figures 4 and 6). The germline-specific expression of other *argonaute* genes, however, varies between morphs. For example, the association of two genes, *Api-piwi6* and *Api-ago3a*, with germline tissue varied substantially among female morphs. *Api-piwi6* expression was germline specific in virginoparae and sexuparae (Figure 5 H–J and Figure S2K, L) but was not specific to germ cells in oviparae (Figure S2O, P). Similarly, *Api-ago3a* was specifically expressed in germ cells in virginoparae (Figure 7 B–E), but showed no germline-specific localisation in either sexuparae or oviparae (Figure S3 A–H). This difference in expression location between the morphs strongly suggests that expressions of *Api-piwi6* and *Api-ago3a* are regulated by the change of photoperiods.

We did observe some inconsistencies between the results of our RT-PCR and *in situ* hybridisation analyses for *Api-ago3b* and *Api-piwi8*. These differences in the detection of expression between the two methods could have been caused by the increased sensitivity of the RT-PCR approach, or because the target tissue for RT-PCR was the whole adult body whilst the *in situ* hybridisation targeted only the dissected ovarioles. This latter explanation would require that these genes be expressed only in non-ovarian tissues.

To our knowledge, this is the first report implicating Piwi proteins in asexual reproduction in a metazoan. However, our results do parallel previous findings obtained for the protozoans *T. thermophila* and *P. tetraurelia*, which resemble aphids in that they alternate between sexual and asexual phases during their life cycle. Like in aphids, protozoan *piwi* genes showed a diversification of expression pattern during sexual and asexual phases of their life cycle. Despite having highly similar structures, these protozoan Piwi proteins show different specificity to distinct classes of small non-coding RNAs [28]–[30]. Functional studies of *piwi* genes must now be conducted in the pea aphid in order to understand the piRNA binding specificities.

Our results indicate that the expansion of genes encoding the Piwi sub-clade of Argonaute proteins in the pea aphid is associated with a diversification of expression profiles that correlates with the different reproductive modes displayed by this insect. Expanded Piwi and Ago3 of the pea aphid belong to the large Argonaute protein family, which also includes the microRNA specific Ago1 and the siRNA specific Ago2 in insects. We previously described a duplication of *ago1* but no duplication of *ago2* in the pea aphid genome [37]. Altogether our results show a global expansion of the Argonaute protein family with the exception of Ago2 in the pea aphid. Beside Argonaute proteins, other components of the microRNA and piRNA pathways are expanded in the pea aphid, such as Dicer-1 (2 copies) and Pasha (4 copies) for the microRNA pathway [37] and Pimet/Hen1 (2 copies) for the piRNA pathway (S. Jaubert-Possamai personal communication). This duplication appears to be specific to the microRNA and piRNA pathways since no duplication of the genes of the siRNA pathway has been identified, suggesting an expansion of a part of several small RNA pathways in the pea aphid genome. The significance of this expansion remains to be understood. A crucial role for Piwi together with Dicer-1 and a protein named Fragile X Mental Retardation Protein (FMRP) has been proposed in germline fate determination during *Drosophila* embryogenesis [38]. In the pea aphid we have identified five copies of *fmr*, suggesting that duplication of *Api-fmr*, together with the duplicated *Api-piwi* and *Api-dicer-1*, may be critical to the specification of germ cells (S. Jaubert-Possamai unpublished data). Further functional analyses will be necessary to clarify the role of these expanded small RNAs machineries in the pea aphid *A. pisum*.

## Materials and Methods

### Pea aphid clones and induction of sexual reproduction

The LSR1-A1-G1 clone of the pea aphid *A. pisum* [22] was reared on broad bean (*Vicia fabae*) at 18°C. Parthenogenetic reproduction was maintained at 16 hours (h) of light and aphids were reared at low density (1 to 5 individuals per plant). Production of sexual morphs was obtained by rearing aphids at 12 h light and 18°C for two generations [39]. Total RNA was extracted by using the RNeasy kit (QIAGEN) from each of the *A. pisum* reproductive morphs: adult parthenogenetic females reared under long day photoperiod (called virginoparae) and producing parthenogenetic clones, adult parthenogenetic females reared under short day photoperiod (called sexuparae) and producing males and sexual oviparous female clones, adult sexual female oviparae, and adult sexual males. Parthenogenetic and viviparous pea aphid morphs used for *in situ* hybridisation were from an obligate parthenogenetic clone reared in the laboratory at the National Taiwan University [40].

### Annotation of genes encoding Piwi-type Argonaute Proteins

Homologues of Piwi/Aub and Ago3 were identified by mining the genomic data in the *A. pisum* LSR1 genome (Acyr 1.2 version of the assembly) at AphidBase (www.aphidbase.com). This was performed using the corresponding *D. melanogaster* sequences as bait and *A. pisum* predicted proteins (program BlastP) and genomic scaffolds (program TBlastn) as targets. The hits were included in a preliminary phylogenetic analysis by using Neighbour-Joining (NJ), which allowed an unambiguous identification of homologues. Gene models from prediction programs were checked, resulting in only a few manual curations. All annotated genes are listed in Table S3. Amino acid sequences were then deduced from the curated pea aphid gene models for all the candidate genes. The domain distribution of the deduced *A. pisum* proteins was predicted by using Pfam [41] and Interproscan [42] software. Because *R. prolixus* (another hemipteran) is to date the closest evolutionary parent with a complete genome sequence (which is not yet fully annotated), we performed a similar research of *piwi*-like and *ago3*-like genes in the scaffolds of that species (Blast at NCBI).

### Phylogenetic analysis

Homologues of Piwi/Aub and Ago3 were also identified in other insects using Blast against GenBank; hit sequences were collected to form a file in which we included all similar *A. pisum* genes. Given the relatively large evolutionary distance between the different insect species represented in the data set (and even among copies of *A. pisum*), analysing protein rather than DNA sequences seemed to be most appropriate. The sequences were aligned using T-Coffee [43]. A few of the sequences (from some Diptera species), being incomplete, were discarded. The N-terminal end of the protein sequence, which is poorly conserved among the different *piwi*-like genes and *ago3*-like genes, was removed. An NJ analysis was performed using MEGA, with the pairwise deletion option and Poisson correction [44]. A Maximum Likelihood (ML) phylogeny analysis was also performed with PHYML on the same alignment, testing different models of substitution. Bootstrap support for the nodes was calculated using 100 replicates.

### Gene expression profiling in different reproductive morphs

The expression level of the eight *Api-piwi* and the two *Api-ago3* genes in the various reproductive morphs was compared by semi-quantitative reverse transcription-polymerase chain reaction (RT-PCR). Expression of *Api-piwi* and *Api-ago3* genes in the different morphs was normalised against the gene encoding ribosomal protein 7 (*Ap-rpl7*) as a reference gene [45]. For each morph, three batches of 10 individuals from three independent biological experiments were frozen in liquid nitrogen 48 h after adult moult and used for RNA extraction. The concentration and quality of the extracted RNA was estimated with a NanoDrop (Thermo Scientific). First strand cDNAs were produced from 500 ng total RNA using the SuperscriptIII reverse transcriptase (Invitrogen) and random nonamers (Promega) following the supplier's instructions. DNA contamination was removed by treating RNA extraction products with RNase-free DNAse (Promega). As a negative control, RT-PCR experiments were realised on total RNA without SuperscriptIII reverse transcriptase.

The standard PCR program comprised an initial step of 4 minutes (min) at 94°C, then multiple cycles composed of 2 min at 94°C, 30 seconds (sec) at the annealing temperature and 1 min 30 sec at 72°C, and a final elongation step of 5 min at 72°C. For quantification of the amplification products, the appropriate number of cycles corresponding to the exponential range was defined for every gene. The sequences of PCR primers, the corresponding annealing temperatures and the appropriate number of cycles used to measure expression level of the different *Api-piwi* and *Api-ago3* genes are listed in Table S3. Images of the RT-PCR Sybr-safe (Invitrogen) stained agarose gels were acquired with a G:BOX Imager (Syngene) and quantification of the bands was performed using Image-J (http://rsbweb.nih.gov/ij/). For each batch of each reproductive morph, the ratio between the band intensity of the amplified gene and *Api-rpl7* reference gene was calculated to normalise for initial variations in sample concentration. The data were subjected to an arc-sine transformation for analysis of variance (ANOVA) in order to test for the significance of variation of expression level (p-value >0.05).

### Cloning, amplification, and synthesising riboprobes of *Api-piwi* and *Api-ago3* genes

We cloned, then amplified, partial sequences of *Api-piwi* and *Api-ago3* genes through RT-PCR. PCR cloning of template sequences for synthesising riboprobes were performed for 40 thermal cycles. Complementary DNA (cDNA) templates were reversely transcribed using HiScript I reverse transcriptase (Bionovas) and poly dT_18_ primers from total RNA extracted from dissected ovaries of the adult virginoparae. Gene-specific primers were designed based upon sequences of putative homologues of *Api-piwi* and *Api-ago3* in AphidBase. PCR fragments containing verified sequences were then cloned into the pGEM-T Easy Vector (Promega) for subsequent *in vitro* transcription. Sense and antisense digoxigenin (DIG)-labelled riboprobes for *in situ* hybridisation were synthesised from linearised plasmids containing target sequences using the DIG RNA Labeling Kit (SP6/T7) (Roche). Probes of *vasa* subjected to double *in situ* hybridisation were labelled with fluorescein (FL) using the DIG/FL labeling mix (Roche). Primer sequences, lengths of PCR amplicons, and annealing temperatures of PCR are summarized in Table S4.

### Whole-mount *in situ* hybridisation and microscopy

Dissected ovarioles, which contain developing oocytes and embryos, were fixed in 3.8% formaldehyde in 1× phosphate-buffered saline (PBS) at 4°C overnight. The steps for single and double *in situ* hybridisation followed the protocol described in [40]. The working concentration of each probe, including sense and antisense strands, ranged from 1.5 to 2.0 ng/µl. We optimised the hybridisation temperatures according to the best signal intensity that could be discriminated between experimental (antisense probes) and control (sense probes) groups. The hybridisation temperature of each probe varied (Table S3). In single *in situ* hybridisation, we applied Nitroblue tetrazolium (NBT)/5-bromo-4-chloro-3-indolyl phosphate (BCIP) (Roche) as the substrates for developing *Api-piwi* and *Api-ago3* signals. For double *in situ* hybridisation, we used 4-benzoylamino-2,5 diethoxybenzenediazonium chloride hemi[zinc chloride] (Fast Blue BB) salt/naphthol-AS-MXphosphate (NAMP) (Sigma) to develop signals of *Api-piwi2* but remained NBT/BCIP as the substrates for *Api-piwi6* and *Api-ago3a*. After signals of *piwi* and *ago3a* were developed, *vasa* signals were then generated using the Fast Red substrate (Roche). In order to increase the signal specificity of *Api-piwi6* and *Api-ago3a*, we briefly rinsed the embryos in methanol after signal development with NBT/BCIP. After *in situ* hybridisation, samples were mounted in 70% of glycerol/1× PBS and photographed with a Leica DMR connected to Canon EOS 5D MarkII digital camera (Canon). Morphological characteristics of developmental stages were described according to Miura *et al*. [46] and locations of germ cells were described according to Chang *et al*. [24]

## Supporting Information

Figure S1 **Expression levels of the** ***Api-piwi*** **and** ***Api-ago3*** **genes in the four reproductive morphs.** Expressions of the eight *Api-piwi* and the two *Api-ago3* genes were quantified in the four reproductive morphs of the pea aphid by semi-quantitative RT-PCR. The figure shows agarose gels after electrophoresis of RT-PCR products. Quantification of gene expression was analysed by semi-quantitative PCR with copy specific primers in the four reproductive morphs of *A. pisum*: parthenogenetic virginoparae, parthenogenetic sexuparae, oviparae sexual females and sexual males. The expression of the ribosomal *Api-rpl7* gene was analysed as a reference gene [45]. For each morph, RT-PCR were realised on total RNA extracted from batches (R1, R2, R3) of 10 pooled adults resulting from three independent biological replicates. As a negative control, RT-PCR experiments were realised on each RNA sample without SuperscriptIII reverse transcriptase (-). Primers used to investigate the expression of *Api-piwi4* co-amplified *Api-piwi1*, so only the amplification product corresponding to *Api-piwi4* (*) was considered for gene expression analysis. Abbreviations, R: replicates.
(TIF)

Figure S2 **Comparison of expressions of** ***Api-piwi3*** **and** ***Api-piwi6*** **in sexuparous and oviparous embryos.** Dissected ovarioles were hybridised with DIG-labelled antisense riboprobes of *Api-piwi3* (A–H) and *Api-piwi6* (I–P), respectively. For orientations and morphological characteristics of embryos refer to Figure 3O. Early development: A, B, E, F, I, J, M, N; Mid development: C, G, K, O; Late development: D, H, L, P. Germ cells stained with antisense *Api-piwi3* and *Api-piwi6* riboprobes are highlighted with arrows (with preferential expression) and double arrows (without preferential expression). (**A, E, I, M**) Germaria and segregated oocytes (stage 1). Transcripts of *Api-piwi3* were weakly expressed in the germaria and the oocyte of sexuparae (arrowheads) whilst transcripts of *Api-piwi3* were not detected in oviparae. *Api-piwi6* expression was distributed in germaria and the oocytes of sexuparae and oviparae (arrowheads). (**B, F, J, N**) Embryos with newly-segregated germ cells (stage 6). Transcripts of *Api-piwi3* were almost not identified in the embryos of sexuparae and oviparae. *Api-piwi6* expression was not detected in the embryos of sexuparae but was evenly distributed in those of the oviparae. (**C, G, K, O**) Extension of the germband and limb bud formation (stage 12–13). Transcripts of *Api-piwi3* were not identified in the embryos of sexuparae and oviparae but expression of *Api-piwi6* in sexuparous embryos was preferentially identified in germ cells. *Api-piwi6* transcripts were evenly distributed in the embryos of oviparae. In panel (G), germ cells are not presented in the shown focal plane. (**D, H, L, P**) Germband retraction (stage 17) and completion of germband retraction (stage 18). Transcripts of *Api-piwi3* were almost not identified in the embryos of sexuparae and oviparae. In sexuparae specific expression of *Api-piwi6* was identified in germ cells located in the dorsal region of the embryo. Universal expression of *Api-piwi6* was detected in the embryos of oviparae. Abbreviations: Bc, endosymbiotic bacteria; Dev, development; Hd, head; St, stage. Scale bar, 20 µm.
(TIF)

Figure S3 **Comparison of expressions of** ***Api-ago3a*** **and** ***Api-ago3b*** **in sexuparous and oviparous embryos.** Dissected ovarioles were hybridised with DIG-labelled antisense riboprobes of *Api-ago3a* (A–H) and *Api-ago3b* (I–P), respectively. For orientation and morphological characteristics of embryos refer to Figure 3O. Early development: A, B, E, F, I, J, M, N; Mid development: C, G, K, O; Late development: D, H, L, P. Germ cells stained with antisense *Api-ago3a* and *Api-ago3b* riboprobes are highlighted with arrows (with preferential expression) and double arrows (without preferential expression). (**A, E, I, M**) Germaria and segregated oocytes (stage 1). Transcripts of *Api-ago3a* were identified in the germaria and the oocytes of sexuparae and oviparae embryos (arrowheads) but transcripts of *Api-ago3b* were not detected. (**B, F, J, N**) Embryos with newly-segregated germ cells (stage 6). Expression of *Api-ago3a* was evenly distributed in the embryos of sexuparae and oviparae. Transcripts of *Api-ago3b* were not identified in the embryo of both sexuparae and oviparae. (**C, G, K, O**) Extension of the germband and limb bud formation (stage 12–14). Transcripts of *Api-ago3a* were evenly distributed in the embryos of sexuparae and oviparae. By contrast, transcripts of *Api-ago3b* were not almost identified in both morphs. (**D, H, L, P**) Germband retraction (stage 17) and completion of germband retraction (stage 18). Expression of *Api-ago3a* was evenly distributed in embryos of sexupare and oviparae, but transcripts of *Api-ago3b* were almost undetected in the embryos of both morphs. Abbreviations: Bc, endosymbiotic bacteria; Dev, development; Hd, head; St, stage. Scale bar, 20 µm.
(TIF)

Figure S4 **Double** ***in situ*** **hybridisation of** ***Api-piwi2*** **and** ***vasa*** **in virginoparous embryos.** (**A–D**) Ovarioles hybridised with DIG-labelled antisense riboprobe of *Api-piwi2*; (**E–H**) Ovarioles hybridised with FL-labelled antisense riboprobes of *vasa*; (**I–L**) Ovarioles hybridised with both DIG-labelled *Api-piwi2* and FL-labelled *vasa* riboprobes. Color keys indicating single and double *in situ* signals are highlighted below the figures. Orientation and developmental stages of embryos refer to Figure 3O. Single *in situ* hybridisations show that both *Api-piwi2* and *vasa* were expressed in germaria and oocytes during early development (panels A and E). From mid development onward, *Api-piwi2* (B–D) and *vasa* (F–H) marked germ cells specifically (arrows). Co-localised signals of *Api-piwi2* and *vasa* (I–L) were identified in germaria, oocytes and embryonic germ cells (arrows). Abbreviations: Bc, endosymbiotic bacteria; Dev, development; Hd, head; St, stage. Scale bar, 20 µm.
(TIF)

Figure S5 **Double** ***in situ*** **hybridisation of** ***Api-piwi6*** **and** ***vasa*** **in virginoparous embryos.** (**A–D**) Ovarioles hybridised with DIG-labelled antisense riboprobe of *Api-piwi6*; (**E–H**) Ovarioles hybridised with FL-labelled antisense riboprobes of *vasa*; (**I–L**) Ovarioles hybridised with both DIG-labelled *Api-piwi6* and FL-labelled *vasa* riboprobes. Color keys indicating single and double *in situ* signals are highlighted below the figures. Orientation and developmental stages of embryos refer to Figure 3O. Locations of germ cells are indicated with arrows. Single *in situ* hybridisations show that both *Api-piwi6* and *vasa* were expressed in germaria and oocytes during early development (panels A and E). From mid development onward, transcripts of *Api-piwi6* were preferentially expressed in germ cells but universal expression of *Api-piwi6* could be identified in somatic cells (B–D). Expression of *vasa* remained specific in germ cells (F–H). Panels (I) to (L) show co-localised signals of *Api-piwi6* and *vasa* in germaria, oocytes and embryonic germ cells. Abbreviations: Bc, endosymbiotic bacteria; Dev, development; Hd, head; St, stage. Scale bar, 20 µm.
(TIF)

Figure S6 **Double** ***in situ*** **hybridisation of** ***Api-ago3a*** **and** ***vasa*** **in virginoparous embryos.** (**A–D**) Ovarioles hybridised with DIG-labelled antisense riboprobe of *Api-ago3a*; (**E–H**) Ovarioles hybridised with FL-labelled antisense riboprobes of *vasa*; (**I–L**) Ovarioles hybridised with both DIG-labelled *Api-ago3a* and FL-labelled *vasa* riboprobes. Color keys indicating single and double *in situ* signals are highlighted below the figures. Orientation and developmental stages of embryos refer to Figure 3O. Locations of germ cells are indicated with arrows. Single *in situ* hybridisations show that both *Api-ago3a* and *vasa* were expressed in germaria and oocytes during early development (panels A and E). From mid development onward, expression of *Api-ago3a* was specifically identified in germ cells (B–D); however, in late embryos background staining was also detected (D). As mentioned in Figures S4 and S5, specific expression of *vasa* was used to mark germ cells. Panels (I) to (L) show co-localised signals of *Api-ago3a* and *vasa* in germaria, oocytes and embryonic germ cells. Abbreviations: Bc, endosymbiotic bacteria; Dev, development; Hd, head; St, stage. Scale bar, 20 µm.
(TIF)

Table S1 Summary of *in situ* hybridisation of *Api-piwi* and *Api-ago3* genes during embryogenesis.
(DOC)

Table S2 (DOC)

Table S3 Primers used for semi quantitative RT-PCR.
(DOC)

Table S4 Primer pairs used for the synthesis of sense and antisense riboprobes.
(DOC)

Data S1 **Alignment of deduced protein sequences of the eight** ***Api-piwi*** **genes and the two** ***Api-ago3*** **genes.** Deduced protein sequence of the eight Api-Piwi and the two Api-Ago3 proteins were aligned with their *D. melanogaster* orthologues Dme-Piwi, Dme-Aub and Dme-Ago3 by using ClustalW2 (http://www.ebi.ac.uk/Tools/msa/clustalw2/). The PAZ and PIWI domains [47] of each protein were deduced from protein sequence by Interproscan [42]. Amino acids (AA) that belong to the PAZ domain are underlined in yellow, AA that belong to the MID domain are underlined in blue and those that belong to the PIWI domain are underlined in gray. Key residues predicted in the PAZ domain to be involved in the binding of sRNA are indicated in blue, those in the MID domain predicted to be involved in anchoring the 5′ phosphate are indicated in pink. The DDH triad of the PIWI domain involved in slicer activity is indicated in red.
(DOC)
