# Supplementary figures and images for: Expansion of Genes Encoding piRNA-Associated Argonaute Proteins in the Pea Aphid: Diversification of Expression Profiles in Different Plastic Morphs
Source: PLoS One. 2011 Dec 5;6(12):e28051. doi: 10.1371/journal.pone.0028051 (PMC3230593; doi:10.1371/journal.pone.0028051)

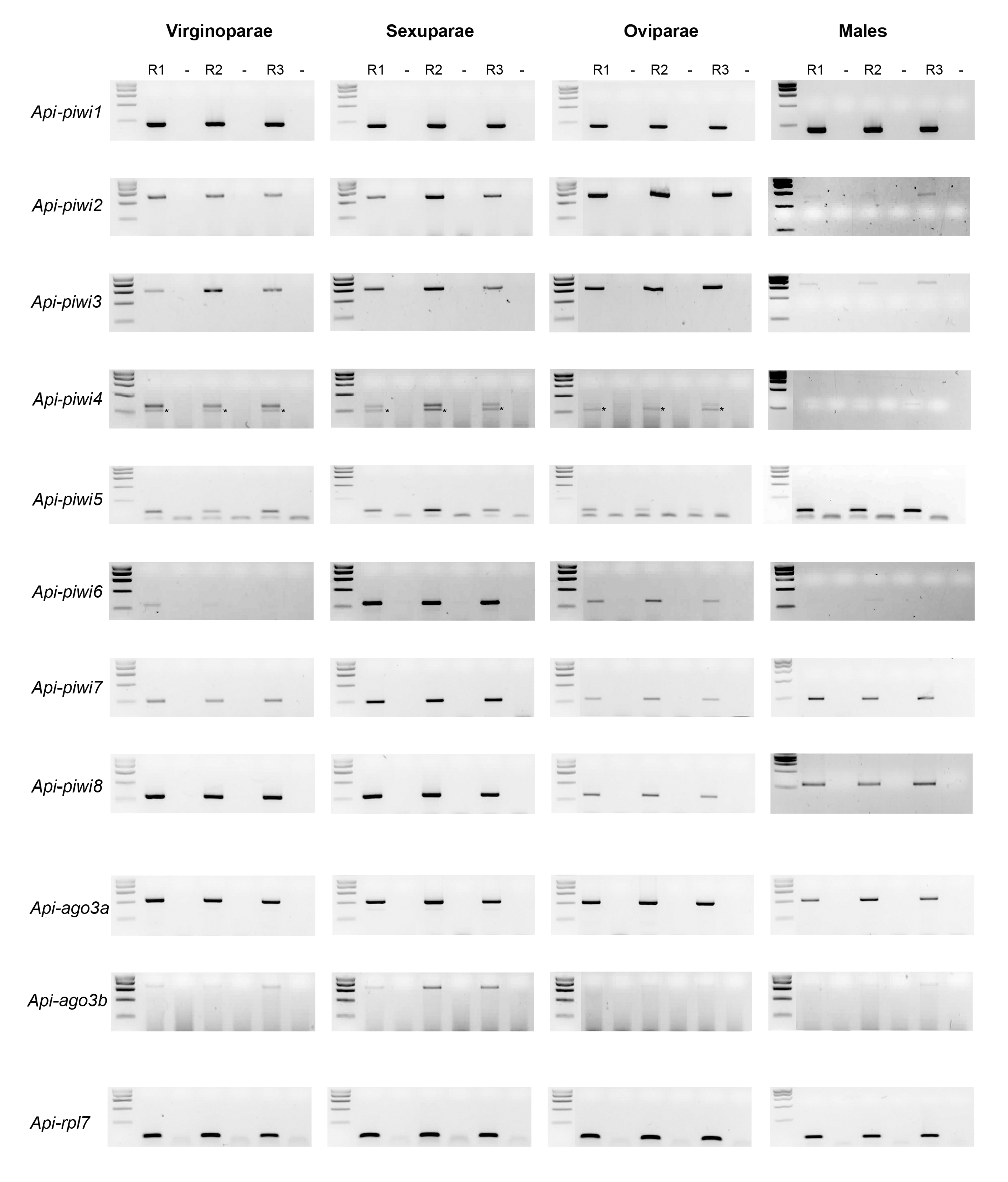

Supplement: Figure S1 — Expression levels of the Api-piwi and Api-ago3 genes in the four reproductive morphs. Expressions of the eight Api-piwi and the two Api-ago3 genes were quantified in the four reproductive morphs of the pea aphid by semi-quantitative RT-PCR. The figure shows agarose gels after electrophoresis of RT-PCR products. Quantification of gene expression was analysed by semi-quantitative PCR with copy specific primers in the four reproductive morphs of A. pisum: parthenogenetic virginoparae, parthenogenetic sexuparae, oviparae sexual females and sexual males. The expression of the ribosomal Api-rpl7 gene was analysed as a reference gene [45]. For each morph, RT-PCR were realised on total RNA extracted from batches (R1, R2, R3) of 10 pooled adults resulting from three independent biological replicates. As a negative control, RT-PCR experiments were realised on each RNA sample without SuperscriptIII reverse transcriptase (-). Primers used to investigate the expression of Api-piwi4 co-amplified Api-piwi1, so only the amplification product corresponding to Api-piwi4 (*) was considered for gene expression analysis. Abbreviations, R: replicates. (TIF) [file pone.0028051.s001.tif]

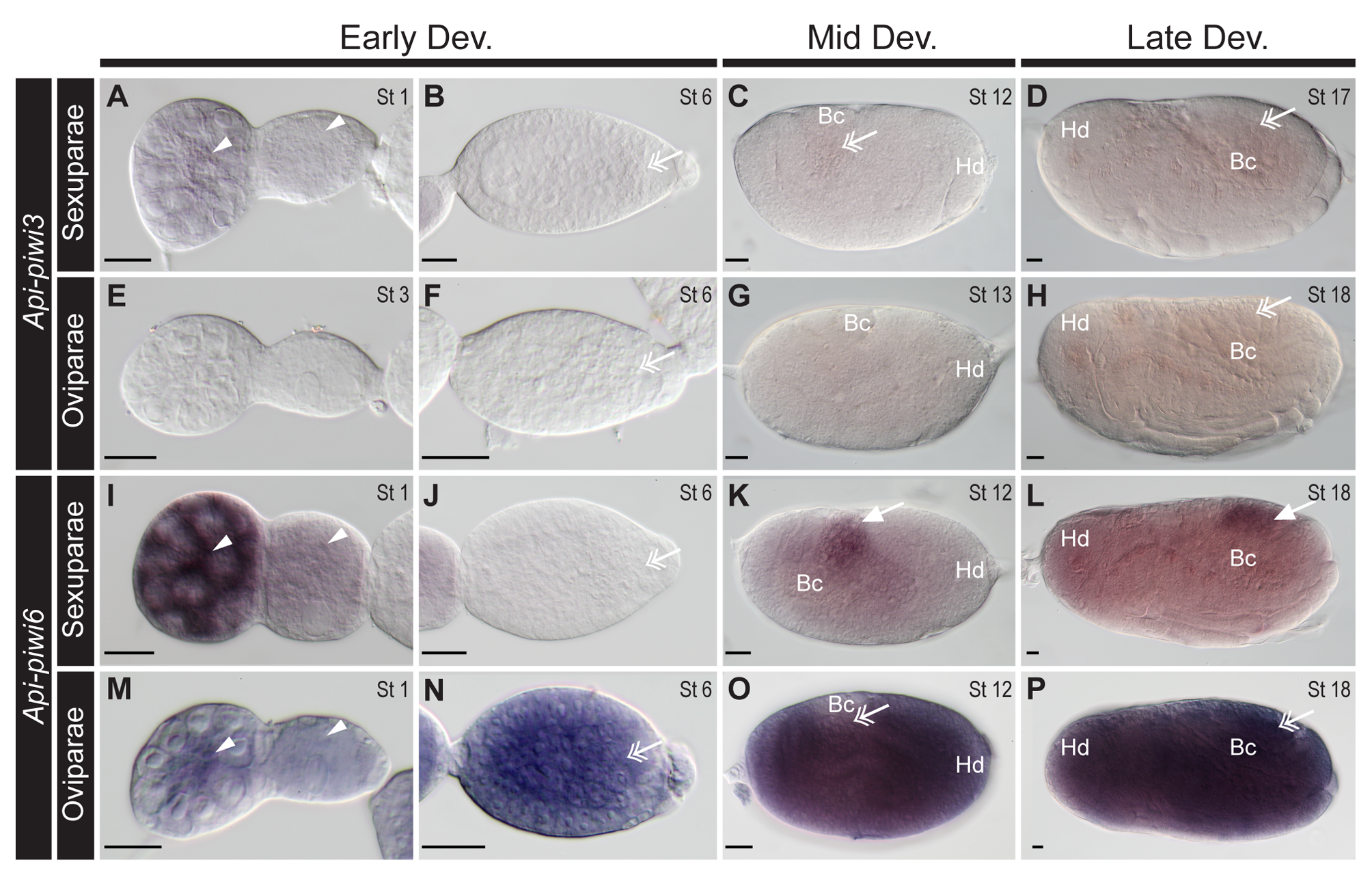

Supplement: Figure S2 — Comparison of expressions of Api-piwi3 and Api-piwi6 in sexuparous and oviparous embryos. Dissected ovarioles were hybridised with DIG-labelled antisense riboprobes of Api-piwi3 (A–H) and Api-piwi6 (I–P), respectively. For orientations and morphological characteristics of embryos refer to Figure 3O. Early development: A, B, E, F, I, J, M, N; Mid development: C, G, K, O; Late development: D, H, L, P. Germ cells stained with antisense Api-piwi3 and Api-piwi6 riboprobes are highlighted with arrows (with preferential expression) and double arrows (without preferential expression). (A, E, I, M) Germaria and segregated oocytes (stage 1). Transcripts of Api-piwi3 were weakly expressed in the germaria and the oocyte of sexuparae (arrowheads) whilst transcripts of Api-piwi3 were not detected in oviparae. Api-piwi6 expression was distributed in germaria and the oocytes of sexuparae and oviparae (arrowheads). (B, F, J, N) Embryos with newly-segregated germ cells (stage 6). Transcripts of Api-piwi3 were almost not identified in the embryos of sexuparae and oviparae. Api-piwi6 expression was not detected in the embryos of sexuparae but was evenly distributed in those of the oviparae. (C, G, K, O) Extension of the germband and limb bud formation (stage 12–13). Transcripts of Api-piwi3 were not identified in the embryos of sexuparae and oviparae but expression of Api-piwi6 in sexuparous embryos was preferentially identified in germ cells. Api-piwi6 transcripts were evenly distributed in the embryos of oviparae. In panel (G), germ cells are not presented in the shown focal plane. (D, H, L, P) Germband retraction (stage 17) and completion of germband retraction (stage 18). Transcripts of Api-piwi3 were almost not identified in the embryos of sexuparae and oviparae. In sexuparae specific expression of Api-piwi6 was identified in germ cells located in the dorsal region of the embryo. Universal expression of Api-piwi6 was detected in the embryos of oviparae. Abbreviations: Bc, endosymb [file pone.0028051.s002.tif]

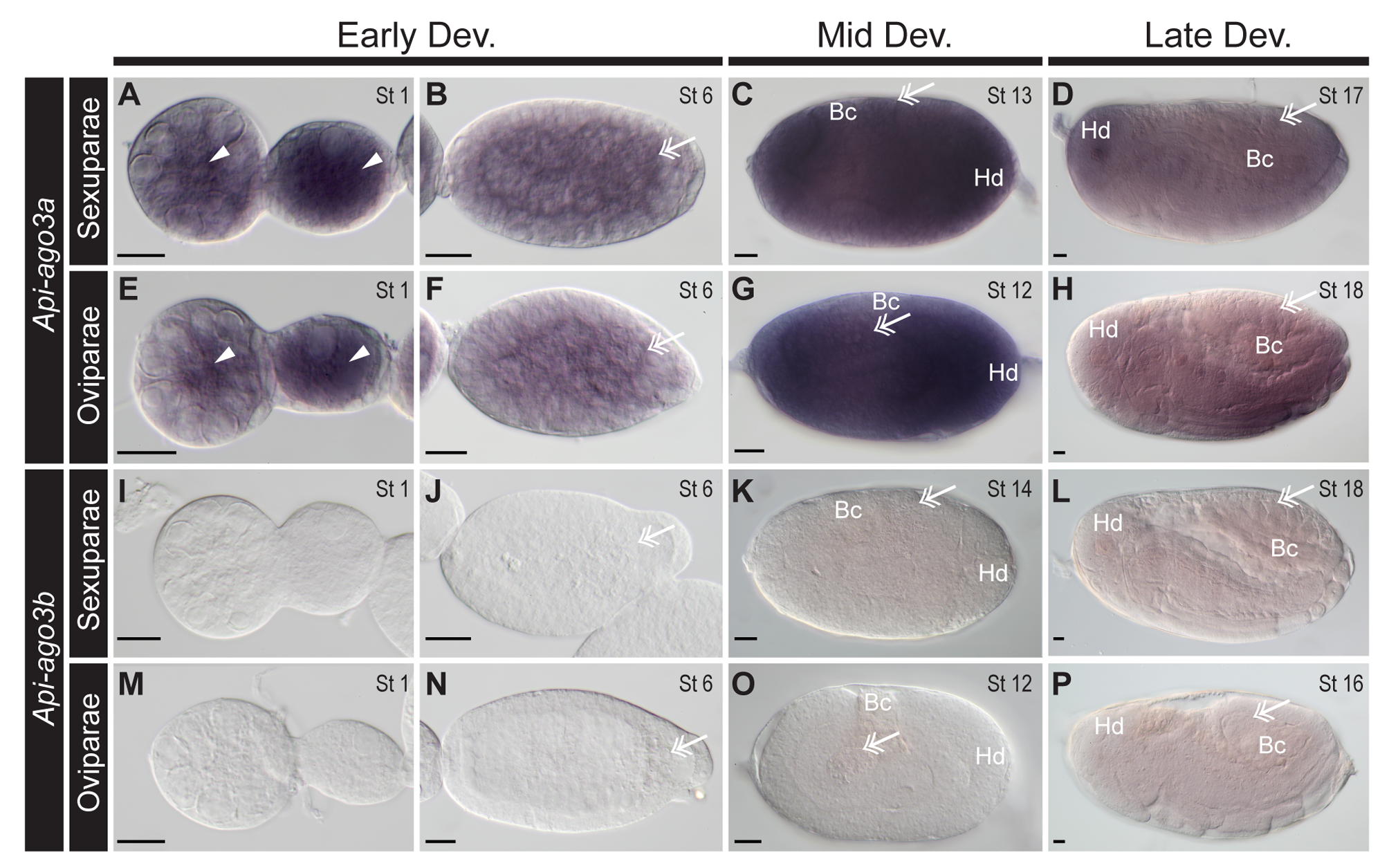

Supplement: Figure S3 — Comparison of expressions of Api-ago3a and Api-ago3b in sexuparous and oviparous embryos. Dissected ovarioles were hybridised with DIG-labelled antisense riboprobes of Api-ago3a (A–H) and Api-ago3b (I–P), respectively. For orientation and morphological characteristics of embryos refer to Figure 3O. Early development: A, B, E, F, I, J, M, N; Mid development: C, G, K, O; Late development: D, H, L, P. Germ cells stained with antisense Api-ago3a and Api-ago3b riboprobes are highlighted with arrows (with preferential expression) and double arrows (without preferential expression). (A, E, I, M) Germaria and segregated oocytes (stage 1). Transcripts of Api-ago3a were identified in the germaria and the oocytes of sexuparae and oviparae embryos (arrowheads) but transcripts of Api-ago3b were not detected. (B, F, J, N) Embryos with newly-segregated germ cells (stage 6). Expression of Api-ago3a was evenly distributed in the embryos of sexuparae and oviparae. Transcripts of Api-ago3b were not identified in the embryo of both sexuparae and oviparae. (C, G, K, O) Extension of the germband and limb bud formation (stage 12–14). Transcripts of Api-ago3a were evenly distributed in the embryos of sexuparae and oviparae. By contrast, transcripts of Api-ago3b were not almost identified in both morphs. (D, H, L, P) Germband retraction (stage 17) and completion of germband retraction (stage 18). Expression of Api-ago3a was evenly distributed in embryos of sexupare and oviparae, but transcripts of Api-ago3b were almost undetected in the embryos of both morphs. Abbreviations: Bc, endosymbiotic bacteria; Dev, development; Hd, head; St, stage. Scale bar, 20 µm. (TIF) [file pone.0028051.s003.tif]

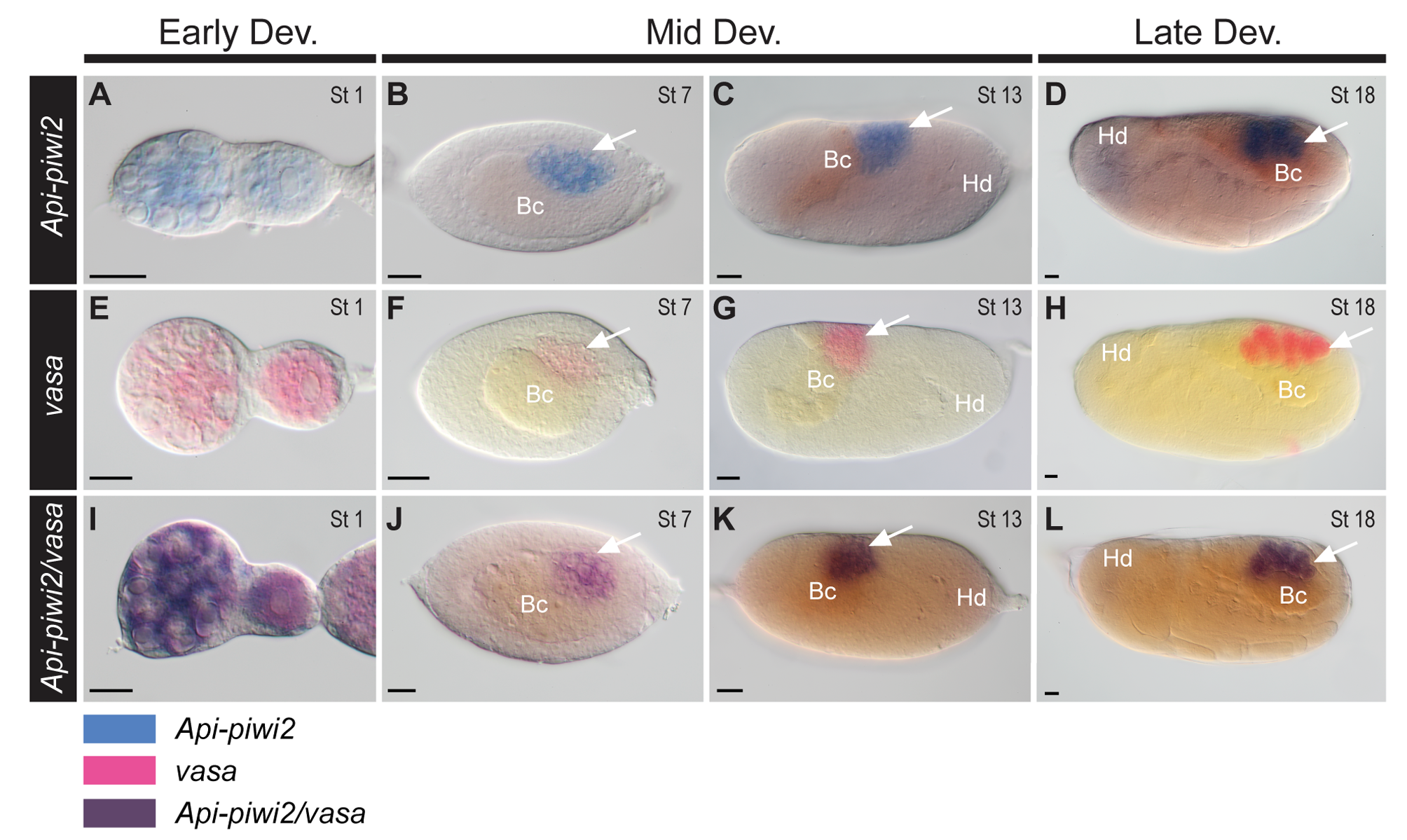

Supplement: Figure S4 — Double in situ hybridisation of Api-piwi2 and vasa in virginoparous embryos. (A–D) Ovarioles hybridised with DIG-labelled antisense riboprobe of Api-piwi2; (E–H) Ovarioles hybridised with FL-labelled antisense riboprobes of vasa; (I–L) Ovarioles hybridised with both DIG-labelled Api-piwi2 and FL-labelled vasa riboprobes. Color keys indicating single and double in situ signals are highlighted below the figures. Orientation and developmental stages of embryos refer to Figure 3O. Single in situ hybridisations show that both Api-piwi2 and vasa were expressed in germaria and oocytes during early development (panels A and E). From mid development onward, Api-piwi2 (B–D) and vasa (F–H) marked germ cells specifically (arrows). Co-localised signals of Api-piwi2 and vasa (I–L) were identified in germaria, oocytes and embryonic germ cells (arrows). Abbreviations: Bc, endosymbiotic bacteria; Dev, development; Hd, head; St, stage. Scale bar, 20 µm. (TIF) [file pone.0028051.s004.tif]

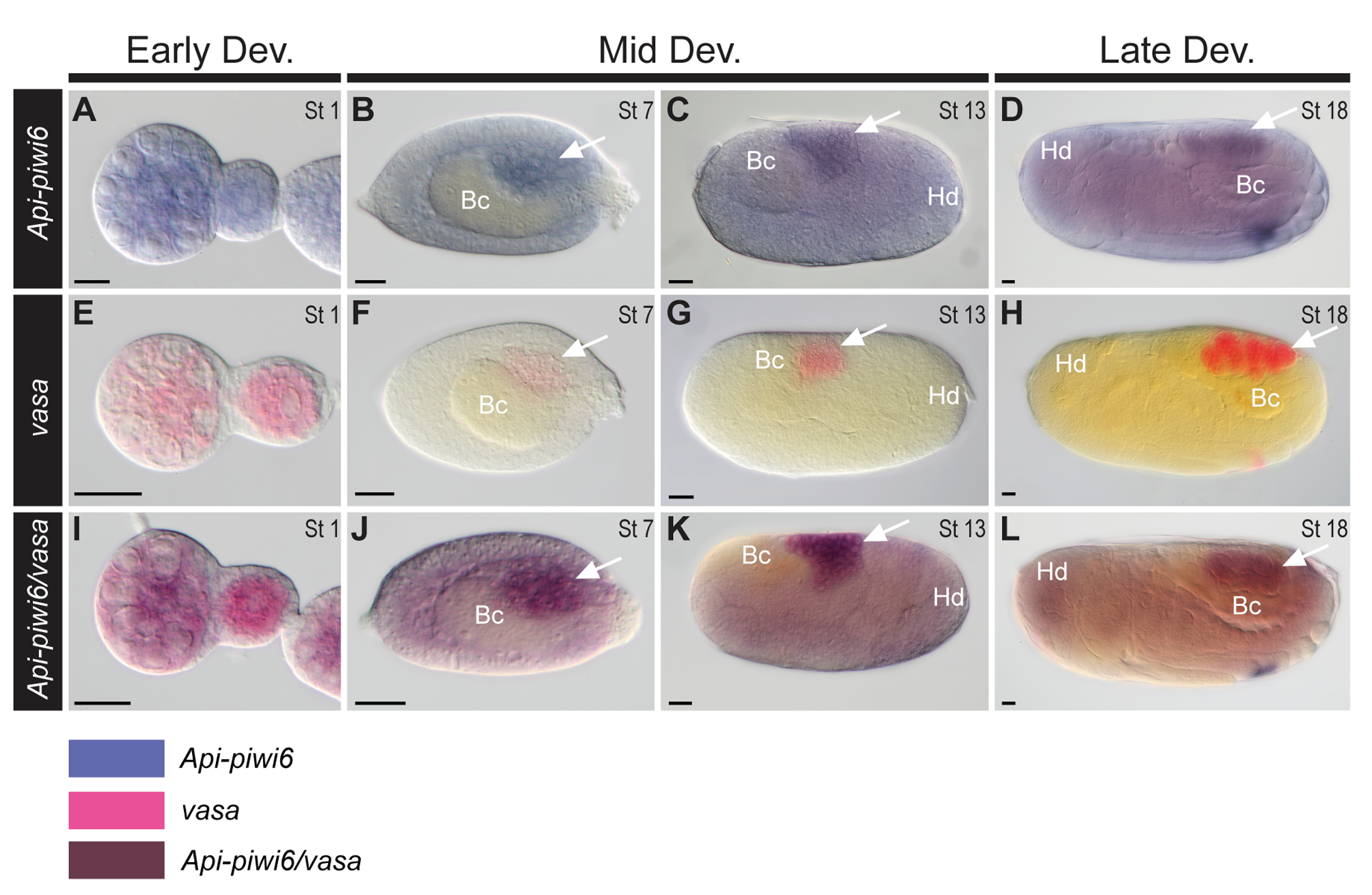

Supplement: Figure S5 — Double in situ hybridisation of Api-piwi6 and vasa in virginoparous embryos. (A–D) Ovarioles hybridised with DIG-labelled antisense riboprobe of Api-piwi6; (E–H) Ovarioles hybridised with FL-labelled antisense riboprobes of vasa; (I–L) Ovarioles hybridised with both DIG-labelled Api-piwi6 and FL-labelled vasa riboprobes. Color keys indicating single and double in situ signals are highlighted below the figures. Orientation and developmental stages of embryos refer to Figure 3O. Locations of germ cells are indicated with arrows. Single in situ hybridisations show that both Api-piwi6 and vasa were expressed in germaria and oocytes during early development (panels A and E). From mid development onward, transcripts of Api-piwi6 were preferentially expressed in germ cells but universal expression of Api-piwi6 could be identified in somatic cells (B–D). Expression of vasa remained specific in germ cells (F–H). Panels (I) to (L) show co-localised signals of Api-piwi6 and vasa in germaria, oocytes and embryonic germ cells. Abbreviations: Bc, endosymbiotic bacteria; Dev, development; Hd, head; St, stage. Scale bar, 20 µm. (TIF) [file pone.0028051.s005.tif]

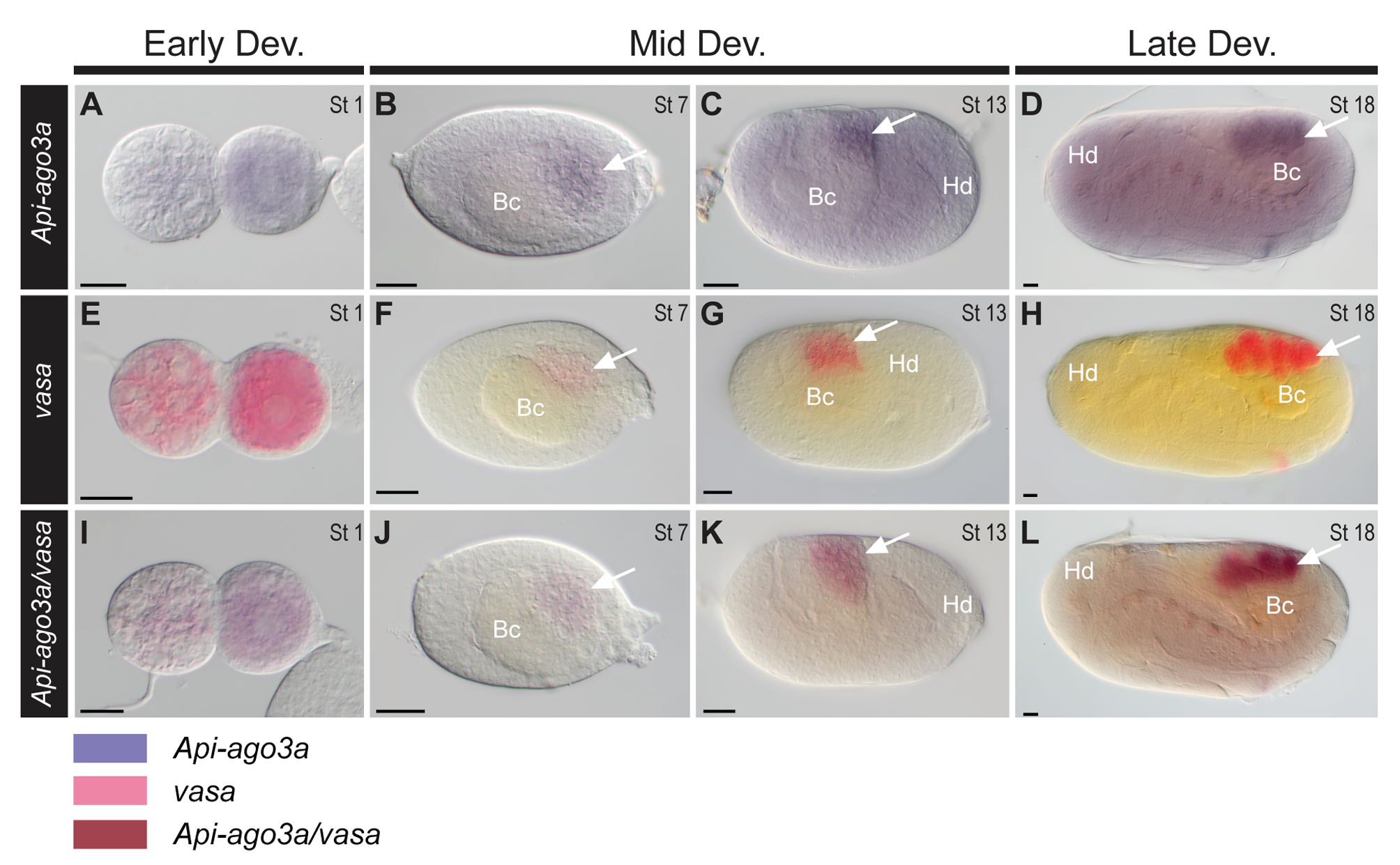

Supplement: Figure S6 — Double in situ hybridisation of Api-ago3a and vasa in virginoparous embryos. (A–D) Ovarioles hybridised with DIG-labelled antisense riboprobe of Api-ago3a; (E–H) Ovarioles hybridised with FL-labelled antisense riboprobes of vasa; (I–L) Ovarioles hybridised with both DIG-labelled Api-ago3a and FL-labelled vasa riboprobes. Color keys indicating single and double in situ signals are highlighted below the figures. Orientation and developmental stages of embryos refer to Figure 3O. Locations of germ cells are indicated with arrows. Single in situ hybridisations show that both Api-ago3a and vasa were expressed in germaria and oocytes during early development (panels A and E). From mid development onward, expression of Api-ago3a was specifically identified in germ cells (B–D); however, in late embryos background staining was also detected (D). As mentioned in Figures S4 and S5, specific expression of vasa was used to mark germ cells. Panels (I) to (L) show co-localised signals of Api-ago3a and vasa in germaria, oocytes and embryonic germ cells. Abbreviations: Bc, endosymbiotic bacteria; Dev, development; Hd, head; St, stage. Scale bar, 20 µm. (TIF) [file pone.0028051.s006.tif]
