# Supplementary material for: Expansion of Genes Encoding piRNA-Associated Argonaute Proteins in the Pea Aphid: Diversification of Expression Profiles in Different Plastic Morphs
Source: PLoS One. 2011 Dec 5;6(12):e28051. doi: 10.1371/journal.pone.0028051 (PMC3230593; doi:10.1371/journal.pone.0028051)
Supplement: Table S1 — Summary of in situ hybridisation of Api-piwi and Api-ago3 genes during embryogenesis. (DOC) [file pone.0028051.s007.doc]

**Table S1** Summary of *in situ* hybridisation of *Api-piwi* and *Api-ago3* genes during embryogenesis

| **Gene** | **Morphs** | **Expression before stage 6** | | **Expression after stage 6** | |
| --- | --- | --- | --- | --- | --- |
|  |  | Somatic | Germaria | Somatic | Germ-spec. |
| *Api-piwi1,4,7* | Vp | + | + | + | + (pref. exp.) |
|  | Sxp | ND | ND | ND | ND |
|  | Ovp | ND | ND | ND | ND |
| *Api-piwi2* | Vp | - | + | - | + |
|  | Sxp | + | + | + | + (pref. exp.) |
|  | Ovp | + | + | + | + (pref. exp.) |
| *Api-piwi3* | Vp | - | - | + | - |
|  | Sxp | - | + | + | - |
|  | Ovp | - | - | + | - |
| *Api-piwi5* | Vp | + | + | + | - |
|  | Sxp | + | + | + | - |
|  | Ovp | + | + | + | - |
| *Api-piwi6* | Vp | - | + | - | + |
|  | Sxp | - | + | + | + (pref. exp.) |
|  | Ovp | + | + | + | - |
| *Api-piwi8* | Vp | + | - | + | - |
|  | Sxp | ND | ND | ND | ND |
|  | Ovp | ND | ND | ND | ND |
| *Api-ago3a* | Vp | + | + | - | + |
|  | Sxp | + | + | + | - |
|  | Ovp | + | + | + | - |
| *Api-ago3b* | Vp | - | - | - | - |
|  | Sxp | - | - | - | - |
|  | Ovp | - | - | - | - |

+: transcripts that can be detected; -: transcripts that can not be detected; Germ-spec: germline specific; pref. exp.: preferential expression in germ cells; ND: not determined; Vp: virginoparae; Sxp: sexuparae; Ovp: oviparae.
