# Supplementary material for: Expansion of Genes Encoding piRNA-Associated Argonaute Proteins in the Pea Aphid: Diversification of Expression Profiles in Different Plastic Morphs
Source: PLoS One. 2011 Dec 5;6(12):e28051. doi: 10.1371/journal.pone.0028051 (PMC3230593; doi:10.1371/journal.pone.0028051)
Supplement: Table S2 — (DOC) [file pone.0028051.s008.doc]

**Table S2**

| **Piwi-like and Ago3-like genes** | **Gen Bank accession number** |
| --- | --- |
| *Ame-piwi* | NP_001159378 |
| *Nvi-piwi1* | XP_001602384 |
| *Nvi-piwi2* | XP_001607362 |
| *Nvi-piwi3* | XP_001605719 |
| *Cfl-piwi* | EFN67778 |
| *Hsa-piwi1* | EFN83189 |
| *Hsa-piwi2* | EFN77932 |
| *Tca-piwi* | EFA07425 |
| *Phu-piwi* | XP_002431988 |
| *Bmo-piwi* | NP_001098066 |
| *Aae-piwi1* | XP_001652831 |
| *Aae-piwi2* | XP_001663870 |
| *Aae-piwi3* | XP_001653082 |
| *Aae-piwi4* | XP_001663409 |
| *Aae-piwi5* | XP_001663408 |
| *Aae-piwi6* | XP_001657626 |
| *Aga-piwi1* | XP_310187 |
| *Aga-piwi2* | XP_564296 |
| *Cqu-piwi1* | XP_001844068 |
| *Cqu-piwi2* | XP_001844024 |
| *Cqu-piwi3* | XP_001867946 |
| *Cqu-piwi4* | XP_001862491 |
| *Cqu-piwi5* | XP_001867947 |
| *Dme-piwi* | NP_476875 |
| *Dme-aub* | NP_476734 |
| *Nvi-ago3* | XP_001603582 |
| *Ame-ago3* | ACV84372 |
| *Hsa-ago3* | EFN87544 |
| *Cfl-ago3* | EFN67840 |
| *Phu-ago3* | XP_002428948 |
| *Dme-ago3* | NP_001036627 |
| *Aae-ago3* | XP_001652945 |
| *Cqu-ago3* | XP_001847030 |
| *Aga-ago3* | XP_319604 |
| *Ada-ago3* | EFR28589 |
| *Bmo-ago3* | NP_001098067 |
| *Tca-ago3* | XP_968053 |
| *Api-piwi1* | XP_001949248 |
| *Api-piwi2* | XP_001942742 |
| *Api-piwi3* | XP_001949497 |
| *Api-piwi4* | XP_001949517 |
| *Api-piwi5* | XP_001945886 |
| *Api-piwi6* | XP_001945669 |
| *Api-piwi7* | XP_001948406 |
| *Api-piwi8* | XP_001947590 |
| *Api-ago3a* | XP_001949977 |
| *Api-ago3b* | XP_003245599 |
