# Supplementary material for: Expansion of Genes Encoding piRNA-Associated Argonaute Proteins in the Pea Aphid: Diversification of Expression Profiles in Different Plastic Morphs
Source: PLoS One. 2011 Dec 5;6(12):e28051. doi: 10.1371/journal.pone.0028051 (PMC3230593; doi:10.1371/journal.pone.0028051)
Supplement: Table S3 — Primers used for semi quantitative RT-PCR. (DOC) [file pone.0028051.s009.doc]

**Table S3** Primers used for semi quantitative RT-PCR.

| **Gene** | **ACYPI** | **Primer** | **Sequence (5’-3’)** | **Tm** | **cycles** |
| --- | --- | --- | --- | --- | --- |
| ***Api-piwi1*** | ACYPI003869 | Api-piwi1F | AAATTACCAATTCGGCGTTG | 60 | 32 |
|  |  | Api-piwi1R | TTGCCCTGATTGGTTAGGAG | 60 | 32 |
| ***Api-piwi2*** | ACYPI008672 | Api-piwi2F | ACCAGTTACGGGTACAATG | 55 | 32 |
|  |  | Api-piwi2R | TCCTCTTCAGGATTGAAGTCA | 55 | 32 |
| ***Api-piwi3*** | ACYPI004735 | Api-piwi3F | ATGCAACGTCCCAAGACAAC | 55 | 30 |
|  |  | Api-piwi3R | TTGGCATAATCACCGGTC | 55 | 30 |
| ***Api-piwi4*** | ACYPI006961 | Api-piwi4F | AAAGTCGTGCAAATCCTGGT | 50 | 32 |
|  |  | Api-piwi4R | ATTTTTCCATCCAGGTAAGATTT | 50 | 32 |
| ***Api-piwi5*** | ACYPI008719 | Api-piwi5F | TCAGACGTCCGTCAAACATT | 58 | 38 |
|  |  | Api-piwi5R | ATCATTATGCACAGAATTGAATAACA | 58 | 38 |
| ***Api-piwi6*** | ACYPI005423 | Api-piwi6F | CTCACACTGGATTGGACGAG | 60 | 38 |
|  |  | Api-piwi6R | CAAACCCAGCCCCAGTAATA | 60 | 38 |
| ***Api-piwi7*** | ACYPI008448 | Api-piwi7F | CTCCAAAGTCCTGCAAATCC | 60 | 30 |
|  |  | Api-piwi7R | CCCTACATGGCAGTCAACCT | 60 | 30 |
| ***Api-piwi8*** | ACYPI005740 | Api-piwi8F | TATGCGTCATTCCAAATTCGCC | 58 | 32 |
|  |  | Api-piwi8R | GCTTTCCGTGACAGACATCA | 58 | 32 |
| ***Api-ago3a*** | ACYPI008078 | Api-ago3aF | CCCCAAGAACATCCTAAATCTG | 58 | 36 |
|  |  | Api-ago3abR | TGACAATTGAGCCATGTGGT | 58 | 36 |
| ***Api-ago3b*** | ACYPI006249 | Api-ago3bF | GTTTGGCTCAGATCGCTACC | 58 | 38 |
|  |  | Api-ago3abR | TGACAATTGAGCCATGTGGT | 58 | 38 |
| ***Api-rpl7*** | ACYPI010200 | Api-rpl7F | GCGCGCCGAGGCTTAT | 60 | 30 |
|  |  | Api-rpl7R | CCGGATTTCTTTGCATTTCTTG | 60 | 30 |

F: forward primer, R: reverse primer, Tm: melting temperature of primers , cycles: number of quantitative amplification cycles.
