# Supplementary material for: Expansion of Genes Encoding piRNA-Associated Argonaute Proteins in the Pea Aphid: Diversification of Expression Profiles in Different Plastic Morphs
Source: PLoS One. 2011 Dec 5;6(12):e28051. doi: 10.1371/journal.pone.0028051 (PMC3230593; doi:10.1371/journal.pone.0028051)
Supplement: Table S4 — Primer pairs used for the synthesis of sense and antisense riboprobes. (DOC) [file pone.0028051.s010.doc]

**Table S4** Primer pairs used for the synthesis of sense and antisense riboprobes.

| Genes | Primer sequences | Length of riboprobes (nt) | Annealing temperature (°C) | Hybridization temperature (°C) |
| --- | --- | --- | --- | --- |
| *Api-piwi1,4,7* | F: GCGTGTTGTTATTCCTGAAAAGGG | 679 | 55 | 55 |
|  | R: TTTGTGTGCCAGTTGACATAGGG |  |  |  |
| *Api-piwi2* | F: CCACTACAAACACCAGTTACGGG | 250 | 55 | 55 |
|  | R: CATTATCACCAGACACTCCAGGC |  |  |  |
| *Api-piwi3* | F: TAACGATAGAATACAGTT | 336 | 50 | 55 |
|  | R: GCGTTTCTAATGTTCTGTGTTGCC |  |  |  |
| *Api-piwi5* | F: CGATATACTTACTATTGATTAATTGT | 216 | 48 | 55 |
|  | R: CAAACTGTAGGGAAAGGGCTCG |  |  |  |
| *Api-piwi6* | F: CTCACACTGGATTGGACGAG | 249 | 52 | 55 |
|  | R: CAAACCCAGCCCCAGTAATA |  |  |  |
| *Api-piwi8* | F: ATGGAGGTGATAACTCAGAGCCTG | 800 | 55 | 60 |
|  | R: TCGCTTTCCGTGACAGACATC |  |  |  |
| *Api-ago3a* | F: AAGAACCCGAGGAACCACCTACAC | 303 | 48 | 60 |
|  | R: CCACATCTGCTTTTTTATGCTCTGG |  |  |  |
| *Api-ago3b* | F: ACTGATACACCAAAAGTCCCTG | 281 | 48 | 60 |
|  | R: AACATAGCCCTTCTTCCGC |  |  |  |
| *vasa* | F: TCGTTTTGGCTCTTCCGCAGAC | 769 | 55 | 65 |
|  | R: GGGCAGTAGCCATCATATCTCGTC |  |  |  |

F: forward primer, R: reverse primer, nt: nucleotide.
