# Supplementary material for: Expansion of Genes Encoding piRNA-Associated Argonaute Proteins in the Pea Aphid: Diversification of Expression Profiles in Different Plastic Morphs
Source: PLoS One. 2011 Dec 5;6(12):e28051. doi: 10.1371/journal.pone.0028051 (PMC3230593; doi:10.1371/journal.pone.0028051)
Supplement: Data S1 — Alignment of deduced protein sequences of the eight Api-piwi genes and the two Api-ago3 genes. Deduced protein sequence of the eight Api-Piwi and the two Api-Ago3 proteins were aligned with their D. melanogaster orthologues Dme-Piwi, Dme-Aub and Dme-Ago3 by using ClustalW2 (http://www.ebi.ac.uk/Tools/msa/clustalw2/). The PAZ and PIWI domains [47] of each protein were deduced from protein sequence by Interproscan [42]. Amino acids (AA) that belong to the PAZ domain are underlined in yellow, AA that belong to the MID domain are underlined in blue and those that belong to the PIWI domain are underlined in gray. Key residues predicted in the PAZ domain to be involved in the binding of sRNA are indicated in blue, those in the MID domain predicted to be involved in anchoring the 5′ phosphate are indicated in pink. The DDH triad of the PIWI domain involved in slicer activity is indicated in red. (DOC) [file pone.0028051.s011.doc]

Dm-Piwi ----------------------------------------------------------MA 2

Dm-Auber --------------------------------------------------MNLPPNPVIA 10

Ap-Piwi1 ---------------------------------------------------MDPNPPRRG 9

Ap-Piwi7 ---------------------------------------------------MDPNPPRRA 9

Ap-Piwi4 ---------------------------------------------------MDPNPPRRA 9

Ap-Piwi8 ------------------------------------------------------------

Ap-Piwi2 ---------------------------------------------------MEPIRPSRG 9

Ap-Piwi5 ---------------------------------------------------MEPIRPSRG 9

Ap-Piwi3 ------------------------------------------------------------

Ap-Piwi6 MTWCGWRGYVIARWRVAEDDIGMSPLKRFHGLSCWVDENRERVSSERDGERGNPIRPSRG 60

Ap-Ago3a -----------MSGRGFLKSRLAKAASKEPEEPPTPQEHPKSEEPPKLAALPIPYT---- 45

Ap-Ago3b -----------MSGRGFLKSRLAQIATKE---PPKPHENP--EEPSKLAALPIPYFTDTP 44

Dm-Ago3 -----------MSGRGNLLSLFNKNAGNMG------------------------------ 19

Dm-Piwi DDQGRGRRRPLNEDDS-------------------------------------------- 18

Dm-Auber RGRGRGRKPNNVEANRG------------------------------------------- 27

Ap-Piwi1 RGRARGGYPPPPNQIG-------------------------------------------- 25

Ap-Piwi7 RGRARGGYPPPPNQIG-------------------------------------------- 25

Ap-Piwi4 RGRARGGYPPPSNQIG-------------------------------------------- 25

Ap-Piwi8 ------------------------------------------------------------

Ap-Piwi2 KARGRPQFPTIQPGAGLSNLPPQGIRGPRPTIPSQPITSSQPITSSQPIASSQPIASSQP 69

Ap-Piwi5 KTR--AKAPGMAP----------------------------------------------- 20

Ap-Piwi3 ----------------------------------------------------MQRPK--- 5

Ap-Piwi6 KSHGRPDFSTNQPGLSGPSEFSQTPLNQPHLSGPPRLIHSTQLHQTP---VTMERPTRKM 117

Ap-Ago3a EVPVAAISSQTLVAGN-------------------------------------------- 61

Ap-Ago3b KVPVDAIITQTLVNGNQTLVNGNQTLANGNQTIVNGDQTLVNGNQTLVNGHQTLVNGNQT 104

Dm-Ago3 ----KSISSKDHEIDSG------------------------------------------- 32

Dm-Piwi -------------------STSRGSGDGPRVKVFRGSSS--------------------- 38

Dm-Auber ------------------FAPSLGQKSDPSHSEGNQASG--------------------- 48

Ap-Piwi1 ------------------QPRGPPPNQSGQPHGARPAQA--------------------- 46

Ap-Piwi7 ------------------QPRGPPPNQSGQPRGARPAQA--------------------- 46

Ap-Piwi4 ------------------QPGGPPPNQSGQPHGSRPAQT--------------------- 46

Ap-Piwi8 -------------------------------------MT--------------------- 2

Ap-Piwi2 LQTPVTGTMPRPPRPMPPRPTGAPQQRAPRPLGPRPGMSSDNLPRPGMSSDNLPRPGVSG 129

Ap-Piwi5 -------------------PTGITQQRAPRLLGPRLGMH--------------------G 41

Ap-Piwi3 ----------TTP-----RAPGAPQQRAPRP--VRPVVS--------------------- 27

Ap-Piwi6 PPRDHGAPQLRTPGAPQLRTPGAPQLRAPRPHGQRTVVS--------------------- 156

Ap-Ago3a -------------------QQVARGRRAMLANIAQGPVS--------------------- 81

Ap-Ago3b LANGNQTLVNGNQTLVNGNQQVARGRRAMLAKFAQRTVS--------------------- 143

Dm-Ago3 -------------------LDFNNSESSRERLLSSHNIE--------------------- 52

Dm-Piwi ---GDPR---------ADPRIEASRERRALEEAPRR-----EGGP-TERKPWGDQYDYLN 80

Dm-Auber ---GNGGG--------GDAQVGPSIEKSSLSAVQMHK---SEGDPRGSVRGRRLITDLVY 94

Ap-Piwi1 ---PPSRPQARNMPGQSGPQS-VEQVTSGLGNMKTG--DALIPIGRGAVRGRQPIENIYY 100

Ap-Piwi7 ---PPSRPQARNMPGQSGPQS-VEQVTSGLGNMKTG--NALIPIGRGAVRGQQPIENIYY 100

Ap-Piwi4 ---PPSGPQVRNMPGKSGPQS-VEQVTNGIGNMKTE--DAPIPIGRRAVRSRQPIENIYY 100

Ap-Piwi8 ---SQCGPQQ-----EFRPQS-VERVTEQLGSLKT------------------------- 28

Ap-Piwi2 DNVPRPGVSGDNAALKQDPMVGVQQITEGLKTLGAG--DALVPLGRGAVRGRRQIDVEHF 187

Ap-Piwi5 DYVPKPRISVDDLAHIQDPMAEVQQITEGLKNLCAE--AALVPLGRGAMRGRRQIE-EHF 98

Ap-Piwi3 --SERPTVP------KQNPMAGVQQVEEGIRNLTTGGDVPQVPIGRGAARGRRQIDVEHY 79

Ap-Piwi6 --NERPSVP------KLNPMAGVQQVEEGIRNLTTG-DVSLVPIGRGAVRGRRQIDLEHY 207

Ap-Ago3a ---QKLCDTIQSAFSNIDLGDLKNIVRPNTSPEHKKADVVSELLTPPKQPQTTIVDIPAE 138

Ap-Ago3b ---QKLSDTIETPFSNIDLGDLKNNVRPNISPDHKKAEVS-KSLTPLKQPQIQIVDIPAA 199

Dm-Ago3 ---TDLITTLQHVNISVGRGRAR-LIDTLKTDDHTSNQFITSESKENITKKTKGPESEAI 108

Dm-Piwi TRP------AELVSKKGTDGVPVMLQTNFFRLKTKP-EWRIVHYHVEFEPSIENPRVRMG 133

Dm-Auber SRP------PGMTSKKGVVGTHITVQANYFKVLKRP-NWTIYQYRVDFTPDVEATRLRRS 147

Ap-Piwi1 RIE-RPESSKSSDGKLGNGGQDIQLTSNYFPITTYT-DWSLYQYRVDFNPVQDKINIQRG 158

Ap-Piwi7 RIE-RPESSKSSDGKLGRGGQDIMLTSNYFPITTYT-DWSLYQYRVDFNPVQDKINIQRG 158

Ap-Piwi4 RLE-RPESSTSSDGKLGKGGQAIQLTSNYFPITTYT-DWSLYQYRVDFNPVQDRINIKRG 158

Ap-Piwi8 ----------------GSGGQPIQLVTNHFQILTTNPDWSLYQYKVDFNPLQNRINNQRG 72

Ap-Piwi2 RTP-RPQSSLGDIGKQGTSGQPVKLLANYFPITSYT-NWCLYQYRVDFNPEEDRISSKRG 245

Ap-Piwi5 KTPVRPQSSLGVIGKQGTSGQPVKLLANYFPITSYT-NWSLYQYRVDFSPEEDRISTKRG 157

Ap-Piwi3 RTP-RPQSSLGAIGKQGKSGQTVPLLANYFPITSYT-NWCLYQYRVDFNPEEDRISTKKG 137

Ap-Piwi6 RTP-RPESSLGAKGKQGKSGQSVKLLANYFPITSYT-NWCLYQYRVDFSPEDDRISTKKG 265

Ap-Ago3a KEEKTPVICRVLKKSEKDAYHMAQLSSNYIRIKLEE-DKGIYEYRVDFNPPVDVKSARFF 197

Ap-Ago3b KEEKPPVICRVLEKSEKDTYHMAQLSSNYIKIKLEE-DKGIYAYRVDFNPPVDAKRARFL 258

Dm-Ago3 ASENGLFFPDLIYGSKG---SSVNIYCNYLKLTTDE-SKGVFNYEVRFFPPIDSVHLRIK 164

Dm-Piwi VLSNHANLLGSGYLFDGLQLFTTRKFEQEIT-----VLSGKSKLD-IEYKISIKFVGFIS 187

Dm-Auber FLYEHKGILG-GYIFDGTNMFCINQFKAVQDSPYVLELVTKSRAG-ENIEIKIKAVGSVQ 205

Ap-Piwi1 LLSAHKELLG-AYIFDGTMLFSSKKYKPDTL-----ELTSKRNFDDEIVIITIKFTQTIE 212

Ap-Piwi7 LLSAHRELLG-AYIFDGSMLFSSKKYKPDTL-----ELTSKRKFDDKIVIITIKFTQIIE 212

Ap-Piwi4 LLTAHKKLLG-AYIFDGSMLFSSKKYKPATL-----ELASKQNFGDDIVIITIKFTQIIE 212

Ap-Piwi8 LLSAHNDLLG-AYIFDGTLLFSSKKYKPDTL-----ELTSKQKYDNTVVIITITFISIIE 126

Ap-Piwi2 LLAQHRELLG-GYLFDGTMLFSGTRFDPPTF-----ELTSTRRQDDQIVIISVKFTNIIE 299

Ap-Piwi5 LLGQHRELLG-GYLFDGTMLFSGTRYDPSTF-----ELTSTRRQDNQIVMILVKFTNVIE 211

Ap-Piwi3 LLGQHRERLG-GYLFDGTMLFSGSRFDPPVF-----ELTSTRRSDDQIVVITVKFTNVIE 191

Ap-Piwi6 LLSQHRERLG-GYLFDGSMLFSGSRFDPPVF-----ELASTRRSDDQIVVITVKFTNVIE 319

Ap-Ago3a LLNEHRDLFP-VKTFDGTLLYIPKMLPQNVT--------KLVGKLRDESEVTLTITFKRK 248

Ap-Ago3b LVNQHRDLFP-IRIFDGTLLYIPKMLPQN------------------------------- 286

Dm-Ago3 YLNDHKDKLGGTKTFDGNTLYLPILLPNKMT--------VFIS-KAEDVELQIRILYKKK 215

Dm-Piwi CAEPRFLQVLNLILRRSMKGLNLELVGRNLFDPRAKIEIREFKMELWPGYETSIRQHEKD 247

Dm-Auber STDAEQFQVLNLILRRAMEGLDLKLVSRYYYDPQAKINLENFRMQLWPGYQTSIRQHEND 265

Ap-Piwi1 KGDHASIQIFNLLIRKALMNLDLSLVGRNYYDDKAKINIPKHRLQLWPGYETTIAMCDSG 272

Ap-Piwi7 KGDHASIQIFNLLIRKALHNLDLSLVGRNYYDEKAKINIPKHKLQLWPGYETTITMCDSG 272

Ap-Piwi4 KGDHASIQVFNLLIRKALMNLNLSLVGRNYYDDKAKIIIPKHSLQLWPGYETTIAMYDSG 272

Ap-Piwi8 KGDYEFIQILNSLLHRSLVNLNLTLVGRKFCDAEAKISIPKYKLQLWPGFETSIGNHDGG 186

Ap-Piwi2 TGDYANIQVFNLLLRNCLRHLKLTLIGRNFYDPDAKIDMAQHKLQLWPGYETTIGRYEDN 359

Ap-Piwi5 TGDYANIQVFNLLLRNCLRHLKLTMIGRNFYDPDAKIDMAQHKLQLWPGYETTIGRYEDN 271

Ap-Piwi3 TGDYANIQVFNLLLRNCLRHLKLTMIGRNFYDPEAKIDMAQYKLQLWPGYETTIGRYEDN 251

Ap-Piwi6 TGDYANIQVFNLLLRNCLRHLKLTMIGRNFYDQDAKIDMAQYKLQLWPGYEATIGRYEDN 379

Ap-Ago3a NSLRECIHFYNVFLRTIMKILGLVEFGRSFYDENKRILIPEFKLEVWPGYITTIDEFENG 308

Ap-Ago3b ------------------------------------ILIPEFKLEVWPGFMAVIDEFENG 310

Dm-Ago3 EEMRNCTQLYNILFDRVMKVLNYVKFDRKQFDPSRPKIIPLAKLEVWPGYVTAVDEYKGG 275

Dm-Piwi ILLGTEITHKVMRTETIYDIMRRCSH-NPAR---HQDEVRVNVLDLIVLTDYNN**R**T**YR**IN 303

Dm-Auber ILLCSEICHKVMRTETLYNILSDAIR-DSDD---YQSTFKRAVMGMVILTDYNNKT**YR**ID 321

Ap-Piwi1 LLLRSEISTKIMREETVLDFLKECAE-SRNRDPQWMVKFKMGVIGSTVLTRYNNQT**YR**ID 331

Ap-Piwi7 LLLRSEISTKIMREETVLDFLKECAE-SRNRDPQWMMKFKMGVIGSTVLTIYNNQT**YR**ID 331

Ap-Piwi4 LLLRTEISTKIMREETVLDFLKECAE-SRSRDPQWMVKFKMGVVGSTVLTRYNNKT**YR**ID 331

Ap-Piwi8 VFLKTEIYTKYIREETVLDFFKECKE-NEGKNSHWMVQFKMSVIGSEVLNRCDNQT**YI**IN 245

Ap-Piwi2 ILLCAEISTKVMRQETVLDFLNQCAA-DRNRNKDWMINFKSGVVGTTVMTKYNNET**YR**ID 418

Ap-Piwi5 ILLCAEISTKVMRQETVLDFLNQCAT-DRNRNKDWMINFKIGVIGTTVMTKYNNET**YR**ID 330

Ap-Piwi3 ILLCAEISTKVMRQETVLDFLNQCAA-DRNRNRDWMINFKSGVVGTTVMTKYNNET**YR**ID 310

Ap-Piwi6 ILLCAEISTKVMRQETVLDFLNQCAA-DRNRNKDWMMNFIRGVVGTTVMTKYNNET**YR**ID 438

Ap-Ago3a LYMCAEVSHRVLRVQTVLQIMTDIMMNAKTNNTDSKQEIMAALCGSTVITHYNRKT**YR**VD 368

Ap-Ago3b LYMRADVSHRVLRVQTVYQIMTDITMNAKKKNKDIKQEIMAALCGSTVITHYNHKT**YR**VD 370

Dm-Ago3 LMLCCDVSHRILCQKTVLEMLVDLYQQNVEHYQESAR---KMLVGNIVLTRYNN**R**T**Y**KIN 332

**R YR**

Dm-Piwi DVDFGQTPKST**F**SCKGR-DISFVE**Y**YLT**KY**NIRIRDHNQPLLISKNRD**K**--ALK-TNASE 359

Dm-Auber DVDFQSTPLCK**F**KTNDG-EISYVD**Y**YKKR**Y**NIIIRDLKQPLVMSRPTD**K**--NIR-GGNDQ 377

Ap-Piwi1 DVDENSNTQST**F**KKRDGSSISYID**Y**YRE**K**HKINLSNHQQPMLVSKKK-**K**-SFQIEGDECE 389

Ap-Piwi7 DVDENSNTQST**F**KKKDGSSISYIN**Y**YRE**K**HGINLSNHQQPMLVSKKK-**K**-SFKIEGDECE 389

Ap-Piwi4 DVDEKSNTRST**F**KKKDGSSISYID**Y**YRE**K**HKINLSNHQQPMLVSKKK-**K**-SFQIEGDECE 389

Ap-Piwi8 DVVEDYNTKST**F**CKEDGYSTSYID**Y**YKQ**KY**GINLSSYCQPILISQKN E-SFINGGDNSE 303

Ap-Piwi2 DIDENSDPNSE**F**SKKDGSKMTYLQ**Y**YKE**K**WNITIRGGRQPMLISKNK **K**-SVRRFGAEDT 476

Ap-Piwi5 DIDENADPNSE**F**SKKDGSKMTYLQ**Y**YKE**K**WNIKICGGRQPMLISKNK **K**-SVRRFGAEDT 388

Ap-Piwi3 DIDENSDPTSE**F**SKKDGSKMTYIQ**Y**YKE**K**WNLTIRTARQPMLISKNK R-SIRRFGEEDT 368

Ap-Piwi6 DIDENSDPTSE**F**SKKDGSKITYIQ**Y**YKENWNLTIRTTRQPMLISKNK R-SIRRFGEEDT 496

Ap-Ago3a DVDFTMNPLST**F**EQNGV-EVTYKD**Y**YKKM**Y**DVEIKNLQQPMIITKAKK**K**DINRKNPDDLG 427

Ap-Ago3b DVDFTMNPLST**F**ERNGI-EITYKD**Y**YKNM**Y**DVEIKNLTQPMIITKAKK**K**DMISSNANDLS 429

Dm-Ago3 DICFDQNPTCQ**F**EIKTG-CTSYVE**Y**YKQYHNINIKDVNQPLIYSIKKSRGIPAEREN--L 389

**F** **Y KY K**

Dm-Piwi LVV**L**IPELCRVTGLNAEMRSNFQLMRAMSSYTRMNPKQRTDRLRAFNHRLQNTPESVKVL 419

Dm-Auber AIMIIPELARATGMTDAMRADFRTLRAMSEHTRLNPDRRIERLRMFNKRLKSCKQSVETL 437

Ap-Piwi1 LV**YL**VPELCTLTGLT**Q**KMRDNRFLMTDLAVYTRVGPSERINKYNSFINRVLTTPKSAESL 449

Ap-Piwi7 LV**YL**VPELCTLTGLT**Q**RMRDNRFLMTDLAVYTRVGPSERIVKYNSFINRVLTTPKSAESL 449

Ap-Piwi4 LV**YL**VPELCTLTGLT**Q**RMRDNRFLMTDLAVYTKAGPSERICKYNSFINRVLTTPKSVDLL 449

Ap-Piwi8 PV**YL**VPELCSFTGITDAMKKNHYLVADLETQRRVHPSERIGRYKNLINRILTTPKSAESL 363

Ap-Piwi2 LV**YL**VPELCIMTGITDAMRNNFTLMKDMAIHTRVNPKERMERLTNFANRLLSTPESVTEL 536

Ap-Piwi5 LV**YL**VPELCIMTGLTDAMRNNFTLMKDMAIHTRVNPKERVERLTNFANRLLSTPDSVTEL 448

Ap-Piwi3 LI**YL**VPELCLMTGITDAMRNNFTLMKDMAIHTRVNPKERMERLTNFANRLLKTPDSVNEL 428

Ap-Piwi6 LI**YL**VPELCVMTGLNDAMRNNFKLMKDMAIHTRVNPKGRMERLTNFSNRLIKTPDSVNEL 556

Ap-Ago3a ICC**L**VPELCNSTGLTDAMKSDFKLMKVLQNHTLVTPEVRQNAIVEFVNRINAHEVASKKF 487

Ap-Ago3b ICC**L**VPELCNLTGLTEAMKNDNKLMKTLQTYTLVTPEVRQNALVEFVNSINAHEGASKRL 489

Dm-Ago3 QFC**L**IPELCYLTGLRDEVRSDNKLMREIATFTRVSPNQRQMALNKFYENVSNTPAAQEIL 449

**TYL Q**

Dm-Piwi RDWNMELDKNVTEVQGRIIGQQNIVFHNGKVPAGEN-ADWQRHFRDQRMLTTPSDGLDRW 478

Dm-Auber KSWNIELDSALVEIPARVLPPEKILFGNQKIFVCDARADWTNEFRTCSMFKN--VHINRW 495

Ap-Piwi1 SRWNLTLSNKLVTFNGRVLTQETLQGNQIKYPAGNA-ADWTSSLRAAPMFTC--AEIKRW 506

Ap-Piwi7 SQWNLTLSNKLVTFNGRVLTQETLQGNQIKYPAGNA-ADWTSSLRTAPMFTC--AVIKRW 506

Ap-Piwi4 SQWNLTLSNKLVTFNGRVLTQETLQGNQMKYPAGNA-ADWTTTLRSAPMFTC--AGIKRW 506

Ap-Piwi8 KQWNLTLSNKLVTIPCHVLPQQSLQGKNYQFPAGNE-ANWTVHLCKLPMFTC--AEIQRW 420

Ap-Piwi2 KRWNLTLSNKLVELTGRTLQPEPIQSRSKGYNGGEE-ADWTKHLRALPMFTS--ATVKNW 593

Ap-Piwi5 KIWNLTLSNKLVELTGRTLQPEPIQSRSKGYNGGEE-ADWTKHLRSLPMFTS--ATVKNW 505

Ap-Piwi3 KRWNLTLSNKLVELTGRILPPEPIQSRTNGYNGGEE-ADWTKNLRSLPMFTS--AIVKHW 485

Ap-Piwi6 KRWNLTLSNKLVELTGRTLPPEPIQSRTNGYNGGEE-ADWTKNLRSLPMFTS--AIMKHW 613

Ap-Ago3a KDWGLMMRPIPVKMEGPVYKRETILLGSNIRKQVGVNMDWGMDVAKNAMFVA--VNMLNW 545

Ap-Ago3b KDWGLMMRPIPAKMEGPVYKRETILLGSNIRKQVGINMDWGIDVAKNAMFVA--ANVLNW 547

Dm-Ago3 NSWGLSLTNNSNKISGRQMDIEQIYFS-KISVSAGRSAEFSKHAVTNEMLKV--VHLSKW 506

Dm-Piwi AVIAPQRNSHELRTLLDSLYRAASGMGLRIRSPQEFIIYDDRTGTYVRAMD-DCVR-SDP 536

Dm-Auber YVITPSRNLRETQEFVQMCIRTASSMKMNICNPIYEEIPDDRNGTYSQAID-NAAA-NDP 553

Ap-Piwi1 AVIGSQSNGGQVRLFIKTLLTVARKMAFNLPPPEIVDIDISNIRQYLTTLD-QVINQMNP 565

Ap-Piwi7 AVIGSQSNSGQVRLFIKTLLTVAKKMSFNLPSPEIVDIDISNIRQYLTTLD-QVINQMNP 565

Ap-Piwi4 AVIGPQSNGGQVRLFIKTLLTVAKKMSFNLPPPEIVDLDISNMRLYTTTLD-QVINQMNP 565

Ap-Piwi8 VVLGPEENGAEVRQFTNTLLQVAKGMSFNLPQPEIVDLKDTSASTYSTTLD-QVINQMDP 479

Ap-Piwi2 TILAPKDCCREVEAFAQSLSKAAQGMTFVLPRPVIFPMVDGRSNTFLNDLE-KVINESNP 652

Ap-Piwi5 YILAPRDCCREVEVFVQSLLKAAQGMTFLLPRPVISPLVDGKSNTFLINLE-KVINESNP 564

Ap-Piwi3 VILVPREYSQEVDPFVQSLAKAASGMNFTLPKPTVVSMEDGRANSVLTHLE-HITNEHNP 544

Ap-Piwi6 VILAPRDCCREVDLFAQTLVKTAQGMNFTLPKPSIVPMNDGKANTFLSNLE-QVINVSNP 672

Ap-Ago3a AIVYNPRDETTAKSFCKQLVSCGRPLGMEINPPKPIKVQGTNPEVFVSTINSTLKNNSDI 605

Ap-Ago3b AIMYNPRDEGTAKSFCRQLIACGNPLGMEINCPIPIKVEGTNPEVFVSTINRTLKNNSEI 607

Dm-Ago3 IIIHLRNYRQAATSLLDNMKQACESLGMNISNPTMISLDHDRIDAYIQALRRNITMN--T 564

Dm-Piwi KLILCLVPNDNAER**Y**SSI**K-**KRGYVDRAVPT**Q**VVTLKTTKNRS-----LMSIATKIAIQLN 591

Dm-Auber QIVMVVMRSPNEEK**Y**SCI**K**KKRTCVDRPVPS**Q**VVTLKVIAPRQQKPTGLMSIATKVVIQMN 613

Ap-Piwi1 SFILCITNRN--DH**Y**HVI**K-**RQLCVNRAVPS**Q**VVSNRQIE-KNN-----MSVCTKIAIQIN 617

Ap-Piwi7 SFIICIINRN--DH**Y**HVI**K**KRQLCVNRAVPS**Q**VVSNRQIE-KNN-----MSVCTKIAIQIN 617

Ap-Piwi4 SFILCIINRN--DH**Y**NVI**K-**RQLCIDRAVPS**Q**VVLMRQME-KNN-----ISVCTNIVIQIN 617

Ap-Piwi8 SFILCVIPNSPSEH**Y**NLI**K-**RQLCLNRPVPS**Q**IVLLKQMKNKND-----MIMYAKISIKMN 534

Ap-Piwi2 SLILCVIPSARGDI**Y**SMI**K-**RKLCIDRAVPS**Q**VVLLKNVQ-KNN-----LSVCTKIAIQIN 706

Ap-Piwi5 SLILCVIPSARGDI**Y**SMI**K-**RKLCIDRAVPS**Q**VVLLKNVQ-KNN-----LSVCTKIAIQIN 618

Ap-Piwi3 ALILCIIQSPRGDI**Y**SLI**K-**RKLCIDRAVPS**Q**VVLLKNVK-KRD-----MSVCTKIAIQIN 598

Ap-Piwi6 AFILCVIPSARGDI**Y**SLI**K-**RKLCIDRAVPS**Q**VVLLKNVQ-KND-----LSICTKIAIQIN 726

Ap-Ago3a QIVVIIFPNQREDR**Y**NAV**K-**RICCSEIGIPS**Q**VIVSRTLSKPER----LQSITQKIALQIN 661

Ap-Ago3b QLVVIMVPNRREDR**Y**NAV**K-**RICCSEIGIPS**Q**VVVSSTLSKPDR----LQSITQKIALQIN 663

Dm-Ago3 QMVVCICHNRRDDR**Y**AAI**K-**KICCSEIPIPS**Q**VINAKTLQNDLK----IRSVVQKIVLQMN 620

**Y K Q**

Dm-Piwi CKLGYTPWMIELPLSGLMTIGF**D**IAKSTRDRKRAYGALIASMDLQQNSTYFSTVTECSAF 651

Dm-Auber AKLMGAPWQVVIPLHGLMTVGF**D**VCHSPKNKNKSYGAFVATMDQKESFRYFSTVNEHIKG 673

Ap-Piwi1 CKLGGAPWRVVIPEKNMMIVGF**D**VCHDKQNKNKSYGALVATMN-NSHTAYFSCVQPHESG 676

Ap-Piwi7 CKLGGAPWRVVIPEKGMMIIGF**D**VCHDKQNKNKSYGALIATMN-DSHTAYFSCVQPHESG 676

Ap-Piwi4 CKLGGAPWRVVIPEKGMMIVGF**D**VCHDKQNKNKSYGALVATMN-DSHTAYFSCVQPHESG 676

Ap-Piwi8 CKLGGAPWRVVIPEKSMMIVGF**D**VCHGK--RNKSYGALIATMN-DTYTSYFSCVQKYESR 591

Ap-Piwi2 CKLGGAPWLVTIPKKGMMIVGF**D**VCHDSQRKNISFGALVSTMN-DAHTSYFSCVEPHESG 765

Ap-Piwi5 CKLGGAPWLVTIPKKGMMIVGF**D**VCHDSQRKNISFGALVSTMN-DSHTSYFSCVEPHESG 677

Ap-Piwi3 CKLGGAPWLVTIPKKGMMIVGF**D**VCHDSQRKNISFGALVATMN-DSHTSYFSCVEPHESG 657

Ap-Piwi6 CKLGGAPWLVTIPKKGMMIVGF**D**VCHDSQRKNISFGALVATMN-DFHTSYFSCVEPHQSG 785

Ap-Ago3a CKLGGACWAIDIPLKNTMIVGI**D**VYHEKGKQMSSVVGFVASMDKTFTEWYSVAAMQRSTH 721

Ap-Ago3b CKLGGACWAIDIPLKNTMIVGI**D**VYHEKDKQMSSVVGFVASMDKTFTEWYSVAAMQRSTH 723

Dm-Ago3 CKLGGSLWTVKIPFKNVMICGI**D**SYHDPSNRGNSVAAFVASINSSYSQWYSKAVVQTK-R 679

**D**

Dm-Piwi DVLANTLWPMIAKALRQYQHEHRKLPSRIVFYR**D**GVSSGSLKQLFEFEVKDIIEKLKTEY 711

Dm-Auber QELSEQMSVNMACALRSYQEQHRSLPERILFFR**D**GVGDGQLYQVVNSEVNTLKDRLDEIY 733

Ap-Piwi1 QELSSYFAMSIAKALNKYRSINKSLPNSIIIYR**D**GVGDGQLSYVHRTEVDMVKKTCKDFY 736

Ap-Piwi7 QEISSYFAMSIAKALHKYRSINKALPNSIIIYR**D**GVGDGQLSYIHRTEVDMVKKTCKDFY 736

Ap-Piwi4 QELSSYFAMSIAKALNKYRSKNKELPKSIIIYR**D**GVGDGHLSYVHRTEVDMLKKTCKDFY 736

Ap-Piwi8 QELLNNFAMNIAKALNKYKSKNNTLPNSIIIYR**D**GMEDDQLSYVHQIEVDMLKKTCKEFY 651

Ap-Piwi2 EELSVHFATGISKALAKYRAKNGALPTSIIVYR**D**GVGEGQISHVHKTEVRLLQTACEQFY 825

Ap-Piwi5 EELSVHFATGISKALNKYRCKNGKLPTSIVVYR**D**GVGEGQISHVQKTEVRLMQTACEKFY 737

Ap-Piwi3 EELSVHFATGITKALAKYRAKNGSLPTSIIVFR**D**GVGEGQISHVHKTEVKLLQTACEQAY 717

Ap-Piwi6 EELSVNFATAITKALAKYRAKNGSLPTSIIVYR**D**GVGEGQISHVHKTEVKLLQTACEQAY 845

Ap-Ago3a QELMKSMQDAFHKVVTQFKLKNGLLPEKIIIYR**D**GVSDGDLKQVEEIELSDLIESFKSYP 781

Ap-Ago3b QELMKSVQDAFHKVVMQFKAKNGLLPEKIIIYR**D**GVSDGDLKQVEEIELRDLIESFKYHP 783

Dm-Ago3 EEIVNGLSASFEIALKMYRKRNGKLPTNIIIYR**D**GIGDGQLYTCLNYEIPQFEMVCGNR- 738

**D**

Dm-Piwi ARVQLSP-PQLAYIVVTRSMNTRFFLNG----QNPPPGTIVDDVITLPERYDFYLVSQQV 766

Dm-Auber KSAGKQEGCRMTFIIVSKRINSRYFTGH----RNPVPGTVVDDVITLPERYDFFLVSQAV 789

Ap-Piwi1 G----ENKIGMAFIIVKKRISTRFFCSNPTNYQNPPPGTVIDNTVTDPTMYDFYLVSQHV 792

Ap-Piwi7 G----ENKIGMAFIIVKKRISTRFFCGNPKNYQNPPPGTVIDNTVTDPTMYDFYLVSQHV 792

Ap-Piwi4 G----EKKVGMAFIIVKKGISTRFFCSNPKNYQNPPPGTVIDNTVTDPSMYDFYLVSQHV 792

Ap-Piwi8 R----EKKVGLAFVIVKISNNTKFFCNNHNTYQNPPPGTVIDNTVTDPTMYDFYLISQNI 707

Ap-Piwi2 G----ASSVPLAFVIVTKRISARFFAPSNRGPENPRPGTIIDSVVTDPTKYDFFLVSQHV 881

Ap-Piwi5 G----ASSVPFAFVIVTKRISARFFAPSNRGPENPRPGTIIDGIVTDPTKYDFFLVSQNV 793

Ap-Piwi3 G----PKSVPFAFVIVTKRISARFFAPSKRGMENPRPGTIIDTVVTDPTKYDFFLVSQHV 773

Ap-Piwi6 G----PKSVPFAFVIVTKRISARFFAPSKRGMENPRPGTIIDTVVTDPTKYDFFLVSQHV 901

Ap-Ago3a G----QYNPMVSLIIVQKRINTRVFQYVNEKYSNPSSGTVIDNTVTRRNYFDFFLVSQHV 837

Ap-Ago3b E----NYNPMVTLIIVQKRISTRVYQYLDEKYSNPSPGTVIDNTVTRRNYFDFFLVSQHV 839

Dm-Ago3 --------IKISYIVVQKRINTRIFSGSGIHLENPLPGTVVDQHITKSNMYDFFLVSQLV 790

Dm-Piwi RQGTVSPTSYNVLYSSMG------LSPEKMQKLTYKMCHLYYNWSGTTRVPAVCQYAKKL 820

Dm-Auber RIGTVSPTSYNVISDNMG------LNADKLQMLSYKMTHMYYNYSGTIRVPAVCHYA**H**KL 843

Ap-Piwi1 TQGTVTPTHYNVIVDTLNETTTTNITPGIMQKLTYKLTHMYYNWSGTVRVPAPCQLA**H**KL 852

Ap-Piwi7 TQGTVTPTHYNVIVDTLNETTTTNITPGILQKLTYKLTHMYYNWSGTVRVPAPCQLA**H**KL 852

Ap-Piwi4 TQGTVTPTHYNVIVDTLNETTATNITPAVMQKLTYKLTHMYYNWSGTVRVPALCQLA**H**KL 852

Ap-Piwi8 TNNAATPTHYNVIWDTLNETTASNFTPTIVQKLTYKLTHMSYNYSNTQMVPGPCQMARKL 767

Ap-Piwi2 RQGTVTPTHYQVIEDTL------RLPPDIMQRLTFKLTHMYYNWSGTVRVPAPCQLA**H**KL 935

Ap-Piwi5 RQGTVTPTYYQVIEDTL------GLPPDIMQRLTYKLTHMYYNWSGTVRVPAPCQLA**H**KL 847

Ap-Piwi3 RQGTVTPTHYQVIEDTL------RLPPDIMQRLTFKLTHMYYNWSGTVRVPAPCQLA**H**KL 827

Ap-Piwi6 RQGTVTPTHYQVIEDTL------GLPPDIMQRLTFKLTHMYYNWSGTVRVPAPCQLA**H**KL 955

Ap-Ago3a RQGTVNPTHYIVLKNGCN------LSVENIQRLSYKLCHLYYNWCGTVKVPAPVQYA**H**KL 891

Ap-Ago3b RQGTVNPTHYIVLKNGCN------LSVENVQRLSYKLCHLYYNWCGTVKVPAPVQYA**H**KL 893

Dm-Ago3 RQGTVTPTHYVVLRDDCN------YGPDIIQKLSYKLCFLYYNWAGTVRIPACCMYA**H**KL 844

**H**

Dm-Piwi ATLVGTNLHSIPQNALEKKFYYL 843

Dm-Auber AFLVAESINRAPSAGLQNQLYFL 866

Ap-Piwi1 AFLTGQSLQSSANPGLEDLLYFL 875

Ap-Piwi7 AFLTAQSLQSPANPGLEDLLYFL 875

Ap-Piwi4 AFLTGQSLQSRANPGLEDLLYFL 875

Ap-Piwi8 ATFTAESLGGPANPHLEDLLYFM 790

Ap-Piwi2 AFLTGQSLRRSPNIGLDELLYFL 958

Ap-Piwi5 AFLTGQSLRRPSNIGLDELLYFL 870

Ap-Piwi3 AFLTGQTLRRAPHTGLDELLYFL 850

Ap-Piwi6 AFLTGQTLRHAPHTGLDELLYFL 978

Ap-Ago3a AYLIGQNVRQKPSDKLCNSLFFL 914

Ap-Ago3b AYLIGQNVRQMPSDKLCNSLFFL 916

Dm-Ago3 AYLIGQSIQRDVAEALSEKLFYL 867

Xxx : PAZ domain **xx** Hs-AGO1 residues involved in binding of sRNA 3’ end [46]

Xx: Mid domain (Piwi subdomain A) **xx** residues involved in anchoring the 5’ phosphate [46]

Xx : Piwi domain (Piwi subdomain B)**xx**: residues involved in slicer activity
